# Nine new species of the spider family Araneidae (Arachnida, Araneae) from Xishuangbanna, Yunnan, China

**DOI:** 10.3897/zookeys.1072.73345

**Published:** 2021-11-19

**Authors:** Xiaoqi Mi, Shuqiang Li

**Affiliations:** 1 College of Agriculture and Forestry Engineering and Planning, Guizhou Provincial Key Laboratory of Biodiversity Conservation and Utilization in the Fanjing Mountain Region, Tongren University, Tongren 554300, Guizhou, China Tongren University Tongren China; 2 Institute of Zoology, Chinese Academy of Sciences, Beijing 100101, China Institute of Zoology, Chinese Academy of sciences Beijing China

**Keywords:** Morphology, new record, orb-weaver spider, taxonomy

## Abstract

Nine new species of the orb-weaver spider family Araneidae Clerck, 1757 from Menglun Town, Xishuangbanna, Yunnan, China are described: *Acusilastongi***sp. nov.** (♂♀), *Chorizopesyui***sp. nov.** (♂♀), *Chorizopesoidesguoi***sp. nov.** (♂♀), *Deionecheni***sp. nov.** (♀), *D.yangi***sp. nov.** (♂♀), *Hypsosingapulla***sp. nov.** (♂♀), *Mangorabaii***sp. nov.** (♂♀), *M.cephala***sp. nov.** (♂♀) and *Miloniagemella***sp. nov.** (♂♀). The genus *Milonia* Thorell, 1890 is recorded from China for the first time. The previous description of *Chorizopesoideswulingensis* (Yin, Wang & Xie, 1994) from Libo County, Guizhou by Mi and Wang (2018) refers to *Chorizopesoidesannasestakovae***sp. nov.** (♂♀). Diagnostic photos of the habitus and copulatory organs of the new species are provided.

## Introduction

The spider family Araneidae Clerck, 1757 is the third largest family in Araneae, with a total of 3067 species in 177 genera worldwide (WSC 2021). In China, 402 species in 50 genera have been recorded (Li 2020, Yao and Li 2021, Li et al. 2021).

Xishuangbanna Tropical Botanical Garden (XTBG) in Menglun Town lies in Mengla County, Yunnan Province, southwest China. A total of 782 spider species have been recorded from this area through an “All Species Inventory” (Li 2020). The number of Araneidae species of this region continually increases due to ongoing research, e.g. – a taxonomic revision of the orb-weaver genus *Eriovixia* Archer, 1951 from this area indicated 13 species, 7 new to science by Mi and Li (2021). This paper is the second paper of our work on Araneidae from the region. Nine new species of the genera *Acusilas* Simon, 1895, *Chorizopes* O. Pickard-Cambridge, 1871, *Chorizopesoides* Mi & Wang, 2018, *Deione* Thorell, 1898, *Hypsosinga* Ausserer, 1871, *Mangora* O. Pickard-Cambridge, 1889 and *Milonia* Thorell, 1890 are described.

## Materials and methods

All specimens were collected by beating shrubs, fogging, or hand collecting and are preserved in 75% ethanol. Type specimens of new species are deposited in the Institute of Zoology, Chinese Academy of Sciences (IZCAS) in Beijing. The type specimens of *Chorizopesoidesannasestakovae* sp. nov. and comparative material of *Deionelingulata* Han, Zhu & Levi, 2009 are deposited in Tongren University (TRU). The specimens were examined with an Olympus SZ51 stereomicroscope. The epigyna were cleared in trypsin enzyme solution for examination and imaging. The left male palps were dissected in ethanol for examination, description, and imaging. Photos of the habitus and copulatory organs were taken with a Kuy Nice CCD mounted on an Olympus BX53 compound microscope. Compound focus images were generated using Helicon Focus v. 6.7.1.

All measurements are given in millimeters. Leg measurements are given as: total length (femur, patella + tibia, metatarsus, tarsus). References to figures in the cited papers are listed in lowercase (fig. or figs); figures in this paper are noted with an initial capital (Fig. or Figs). Abbreviations used in the text and figures are as follows: ALE anterior lateral eye; AME anterior median eye; BE broken embolus; C conductor; CD copulatory duct; CO copulatory opening; E embolus; ET embolic thorn; FD fertilization duct; MA median apophysis; MOA median ocular area; MP median plate; PLE posterior lateral eye; PME posterior median eye; SA subterminal apophysis; Sc scape; Sp spermatheca; TA terminal apophysis; TE tegular extension.

## Taxonomy

### Family Araneidae Clerck, 1757

#### Genus Acusilas

Taxon classificationAnimaliaAraneaeAraneidae

Simon, 1895

1F00D19C-74F9-5059-A68B-A560A5EED334

Acusilas Simon, 1895: 785; Schmidt and Scharff 2008: 7.

##### Type species

. *Acusilascoccineus* Simon, 1895 from Indonesia

##### Comments

. Nine *Acusilas* species from Asia and one species from Africa are known.

#### Acusilas tongi sp. nov.

Taxon classificationAnimaliaAraneaeAraneidae

07C2A2E8-A856-51C2-A78D-7D4B1DE4E466

http://zoobank.org/9ED4228E-0711-4E28-A95F-8C4FC129B2EC

Figs 1 , 2 , 20A

##### Type material

**. Holotype.** ♂ (IZCAS-Ar42503), China: Yunnan, Xishuangbanna, Mengla County, Menglun Township, Menglun Nature Reserve, primary tropical seasonal rainforest (21°57.43'N, 101°12.28'E, ca 792 m), 19–25. XI.2006, G. Zheng leg. ***Paratypes***: 1♂ (IZCAS-Ar42504), rubber plantation (approx. 20 years old) (21°54.47'N, 101°15.98'E, ca 570 m), 5–12.XI.2006, G. Zheng leg.; 1♀ (IZCAS-Ar42505), rubber plantation (approx. 20 years old) (21°54.65'N, 101°16.26'E, ca 570 m), 5–12.XII.2006, G. Zheng leg.; 1♀ (IZCAS-Ar42506), secondary tropical seasonal moist forest (21°54.61'N, 101°17.01'E, ca 630 m), 28.VII.2007, G. Zheng leg.; 1♂ (IZCAS-Ar42507), low evergreen forest along G213 roadside (21°53.79'N, 101°17.15'E, ca 590 m), 27.XI.2009, G. Tang leg.; 1♀ (IZCAS-Ar42508), Xishuangbanna Tropical Botanical Garden, grapefruit plantation (21°54.07'N, 101°16.36'E, ca 540 m), 22.VII.2018, X. Mi leg.; 1♂ (IZCAS-Ar42509), Xishuangbanna Tropical Botanical Garden, Yulinjiegou scenic spot (21°55.13'N, 101°16.08'E, ca 550 m), 29.VII.2018, X. Mi leg.; 1♀(IZCAS-Ar42510), Teak plantation (21°54.03'N, 101°16.39'E, ca 550 m), 10.VIII.2018, Z. Bai et al. leg.; 1♂ (IZCAS-Ar42511), site 5 around the dump (21°54.37'N, 101°16.07'E, ca 620 m), 6.V.2019, Y. Tong leg.

##### Etymology

. The species is named after Dr. Yanfeng Tong (Shenyang Normal University), one of the collectors of the type specimens; noun (name) in genitive case.

##### Diagnosis

. The new species resembles *A.malaccensis* in habitus but can be distinguished by the: 1) interrupted stripes on the female abdomen vs. uninterrupted (Murphy and Murphy, 1983: fig. 16); 2) spermatheca ovoid vs. globular (Murphy and Murphy, 1983: fig. 12).

##### Description

**Figure 1.**
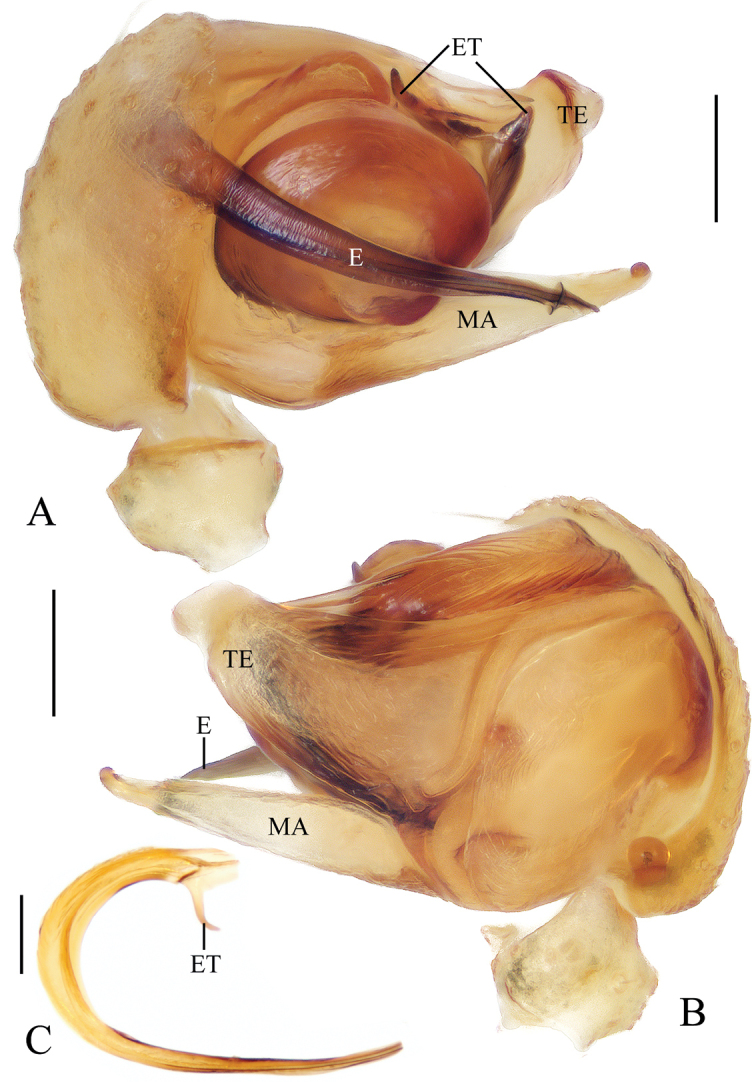
*Acusilastongi* sp. nov., male palp **A, B** holotype **C** broken embolus **A** prolateral view **B** retrolateral view **C** broken embolus in female paratype’s epigyne. Scale bars: 0.1mm

**Figure 2.**
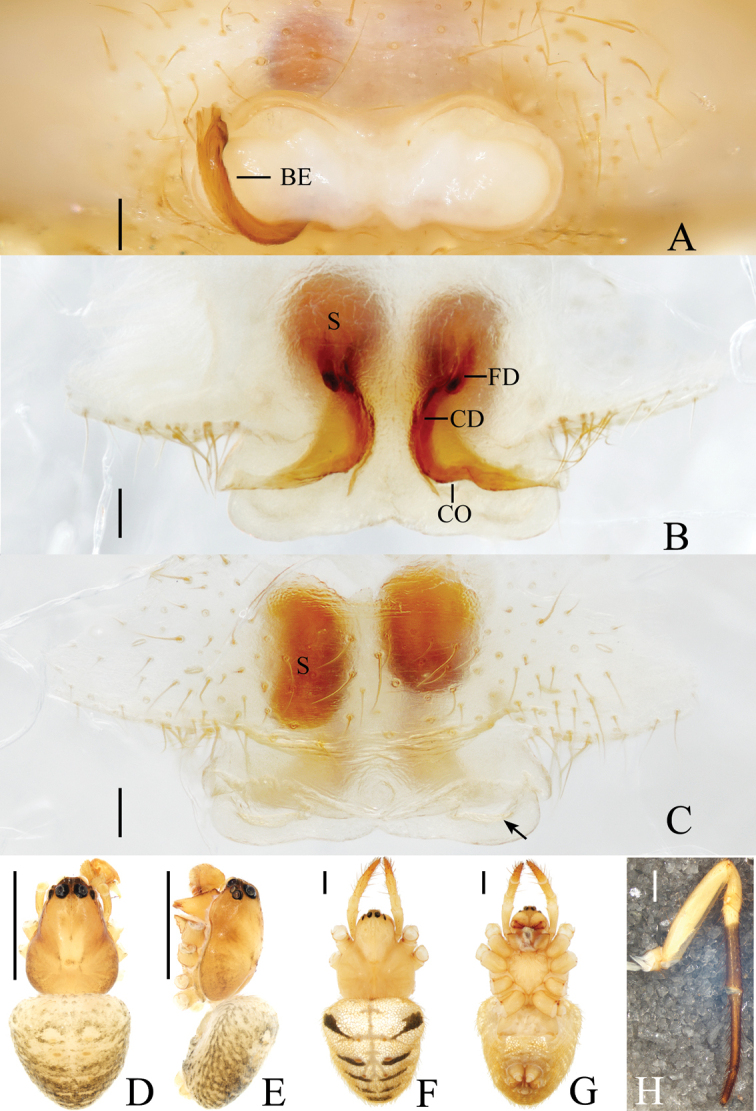
*Acusilastongi* sp. nov. **A–C, F–H** female paratype IZCAS-Ar42505 **D, E** holotype **A** epigyne, ventral view **B** ibid., posterior view **C** ibid., anterior view **D** habitus, dorsal view **E** ibid., lateral view **F** ibid., dorsal view **G** ibid., ventral view **H** left leg I, prolateral view. Scale bars: 0.1mm (**A–C**); 1mm (**D–H**)

**. Male** (holotype, Figs 1A, B, 2D, E, 20A). Total length 2.15. Carapace 1.15 long, 0.90 wide. Abdomen 1.05 long, 1.15 wide. Clypeus 0.08 high. Eye sizes and interdistances: AME 0.13, ALE 0.05, PME 0.10, PLE 0.08, AME-AME 0.08, AME-ALE 0.03, PME-PME 0.10, PME-PLE 0.08, MOA length 0.28, anterior width 0.28, posterior width 0.28. Leg measurements: I 2.75 (0.90, 0.95, 0.50, 0.40), II 2.55 (0.80, 0.85, 0.50, 0.40), III 1.65 (0.55, 0.55, 0.30, 0.25), IV 2.25 (0.75, 0.75, 0.40, 0.35). Carapace pear-shaped, yellowish brown, cervical groove obvious, posterior eyes surrounded with black. Chelicerae yellowish brown, four promarginal teeth, lacking retromarginal teeth. Endites, labium yellow. Sternum yellow, with sparse, dark setae. Legs yellowish brown without annulations. Abdomen blunt anteriorly, pointed posteriorly, about 1.1 times wider than long, dorsum grayish yellow with irregular dark patches; venter grayish yellow with darker spots forming inconspicuous stripes. Spinnerets yellow.

Palp (Figs 1A–C, 20A): median apophysis prominent, about 4/5 length of cymbium; embolus about two times length of cymbium, runs anti-clockwise, curved about 180° from its origin, with two thorns at base (embolic thorn, stipes extended), distal end pointed toward tip of median apophysis.

**Female** (paratype IZCAS-Ar42505, Fig. 2A–C, F–H). Total length 8.20. Carapace 4.20 long, 3.20 wide. Abdomen 5.10 long, 4.40 wide. Clypeus 0.15 high. Eye sizes and interdistances: AME 0.18, ALE 0.13, PME 0.18, PLE 0.15, AME-AME 0.22, AME-ALE 0.10, PME-PME 0.25, PME-PLE 0.18, MOA length 0.55, anterior width 0.55, posterior width 0.55. Leg measurements: I 12.00 (3.60, 4.50, 2.70, 1.20), II 11.30 (3.50, 4.00, 2.60, 1.20), III 7.30 (2.50, 2.60, 1.30, 0.90), IV 11.10 (3.50, 4.00, 2.50, 1.10). Carapace pear-shaped, yellow, cervical groove obvious, posterior eyes surrounded with black. Chelicerae yellow, four promarginal teeth, three retromarginal teeth. Endites and labium yellow. Sternum yellow, with sparse, dark setae. Legs: femur, patella, and basal 1/4 of tibia yellow, remaining 3/4 of tibia, metatarsus, and tarsus dark brown. Abdomen triangular in dorsal view, slightly longer than wide, yellow with nine transverse black stripes; venter yellow with dozens of white spots medially.

Epigyne (Fig. 2A–C): wider than long, with narrow rim anteriorly and laterally, concave anteriorly; posterior lip hooked (see arrow in Fig. 2C); copulatory openings narrow, located posteriorly; copulatory ducts shorter than spermatheca; spermathecae oval.

##### Variation

. Total length: ♂♂ 1.75–2.25; ♀♀ 8.2–10.80.

##### Distribution

. China (Yunnan).

#### Genus Chorizopes

Taxon classificationAnimaliaAraneaeAraneidae

O. Pickard-Cambridge, 1871

7A29B7EB-1FD1-5836-8256-4715DF175067

Chorizoopes O. Pickard-Cambridge, 1871: 738; Tikader 1982: 157; Kallal and Hormiga 2019: 473

##### Type species.

*Chorizoopesfrontalis* O. Pickard-Cambridge, 1871 from SriLanka

##### Comments

. A total of 29 species of *Chorizopes* are known from China, India, Pakistan, Sri Lanka, Myanmar, Korea, Japan, and Madagascar. Illustrations indicate that the following species may belong to other genera: *C.calciope* (Simon, 1895), *C.kastoni* Gajbe & Gajbe, 2004, *C.khandaricus* Gajbe, 2005, *C.khedaensis* Reddy & Patel, 1993, *C.pateli* Reddy & Patel, 1993, *C.quadrituberculata* Roy, Sen, Saha & Raychaudhuri, 2014, *C.rajanpurensis* Mukhtar & Tahir, 2013, *C.tikaderi* Sadana & Kaur, 1974.

#### Chorizopes yui sp. nov.

Taxon classificationAnimaliaAraneaeAraneidae

3A7B0E84-C9A7-54BA-95AA-3E9FD9B9239C

http://zoobank.org/18204F0A-7C55-4B53-BFC7-3074E9C74E6B

Figs 3 , 4

##### Type material

**. *Holotype***. ♂ (IZCAS-Ar42512), China: Yunnan, Xishuangbanna, Mengla County, Menglun Township, Menglun Nature Reserve, high plantations near G213 roadside (21°54.12'N, 101°16.93'E, ca 590 m), 24.XI.2009, G. Tang leg. ***Paratypes***: 1♂ (IZCAS-Ar42513), *Anogeissusacuminata* plantation (approx. 20 years old) (21°53.99'N, 101°16.81'E, ca 610 m), 19.VIII.2007, G. Zheng leg.; 2♀ (IZCAS-Ar42514–42515), garbage dump, secondary tropical forest (21°54.38'N, 101°16.82'E, ca 620 m), 23.XI.2009, G. Tang leg.; 1♂ (IZCAS-Ar42516), Lüshilin Forest Park, limestone tropical seasonal rainforest (21°54.56'N, 101°16.86'E, ca 610 m), 29.XI.2009, G. Tang leg.; 1♀ (IZCAS-Ar42517), secondary tropical forest, bamboo plantation along G213 roadside (21°53.82'N, 101°16.99'E, ca 610 m), 3.VIII.2018, Z. Bai leg.; 1♂ (IZCAS-Ar42518), Xishuangbanna Tropical Botanical Garden, site 1 around the dump (21°53.28'N, 101°16.75'E, ca 630 m), 25.IV.2019, Z. Bai leg.; 1♀ (IZCAS-Ar42519), Xishuangbanna Tropical Botanical Garden, Baihuayuan (21°55.60'N, 101°14.87'E, ca 540 m), 3.V.2019 night, C. Wang leg.

##### Etymology

. The species is named after Dr. Hao Yu, one of the collectors of the type specimens; noun (name) in genitive case.

##### Diagnosis

. The new species can be distinguished from congeneric species by the: 1) yellowish white abdomen with a dark rhomboid patch; 2) triangular copulatory openings; 3) translucent, thread-like terminal apophysis; 4) fan-shaped median apophysis in prolateral view.

##### Description

**Figure 3.**
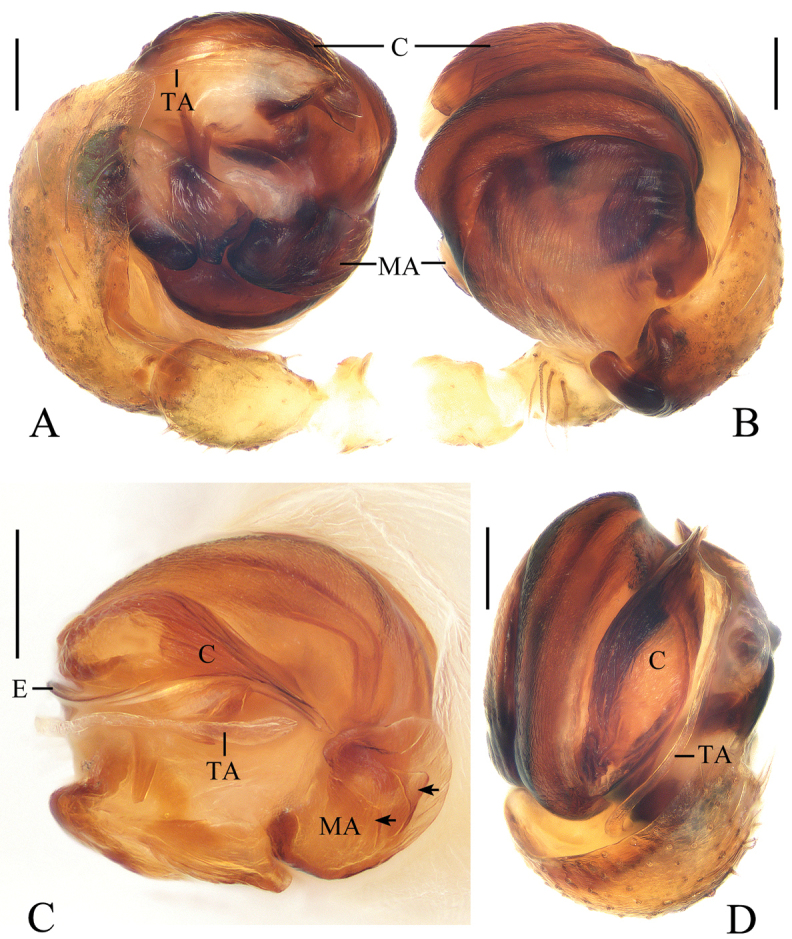
*Chorizopesyui* sp. nov., male palp **A**, **B**, **D** holotype **C** male paratype IZCAS-Ar42513. **A** prolateral view **B** retrolateral view **C** expanded in lactic acid **D** apical view. Scale bars: 0.1mm

**Figure 4.**
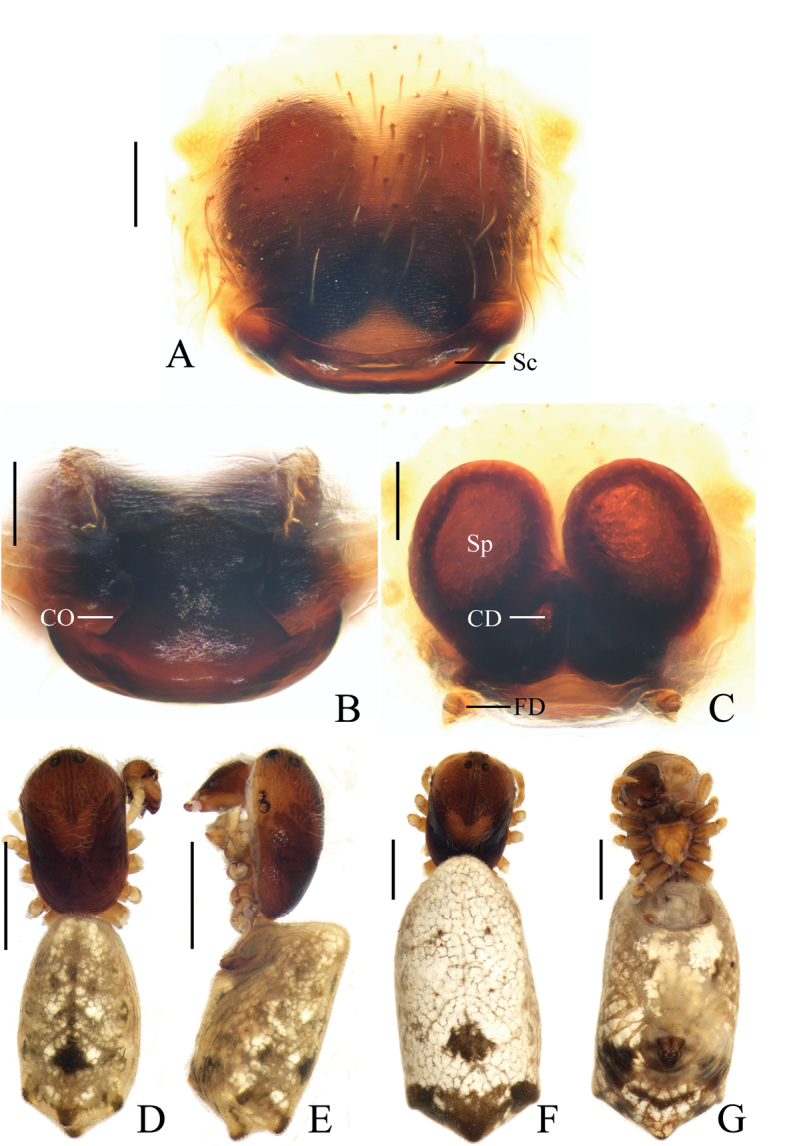
*Chorizopesyui* sp. nov. **A–C**, **F**, **G** female paratype IZCAS-Ar42514 **D**, **E** holotype **A** epigyne, ventral view **B** ibid., posterior view **C** vulva, dorsal view **D** habitus, dorsal view **E** ibid., lateral view **F** ibid., dorsal view **G** ibid., ventral view. Scale bars: 0.1mm (**A–C**); 1mm (**D–G**)

**. Male** (holotype, Figs 3A, B, D, 4D, E). Total length 3.60. Carapace 1.60 long, 1.00 wide. Abdomen 2.00 long, 1.20 wide. Clypeus 0.10 high. Eye sizes and interdistances: AME 0.13, ALE 0.08, PME 0.10, PLE 0.09, AME-AME 0.13, AME-ALE 0.45, PME-PME 0.18, PME-PLE 0.35, MOA length 0.33, anterior width 0.35, posterior width 0.35. Leg measurements: I 3.65 (1.05, 1.30, 0.80, 0.50), II 3.75 (1.05, 1.45, 0.75, 0.50), III 2.25 (0.60, 0.75, 0.45, 0.45), IV 4.15 (1.15, 1.50, 0.90, 0.60). Carapace oval, brown, elevated, cervical groove inconspicuous. Chelicerae brown, seven promarginal teeth. Endites yellow, labium triangular, brown. Sternum triangular, yellowish brown, with pale setae. Legs yellow with brown annulations. Abdomen cylindrical, with pair of lateral tubercles and two vertically arranged tubercles posteriorly, grayish yellow with dark rhomboid patch; venter grayish yellow with large, white patch medially. Spinnerets yellowish brown.

Palp (Fig. 3): paracybium flattened; median apophysis fan-shaped in prolateral view, with two lamellar spurs (see arrows in Fig. 3C); embolus length about equal to bulb diameter, curved, slightly flattened; conductor about 4/5 length of bulb diameter in prolateral view; terminal apophysis translucent, slender, curved, length almost equal to that of embolus.

**Female** (paratype IZCAS-Ar42514, Fig. 4A–C, F, G). Total length 6.50. Carapace 2.10 long, 1.40 wide. Abdomen 4.70 long, 2.60 wide. Clypeus 0.13 high. Eye sizes and interdistances: AME 0.13, ALE 0.08, PME 0.09, PLE 0.10, AME-AME 0.20, AME-ALE 0.53, PME-PME 0.25, PME-PLE 0.73, MOA length 0.40, anterior width 0.43, posterior width 0.43. Leg measurements: I 4.70 (1.35, 1.70, 1.05, 0.60), II 4.60 (1.35, 1.65, 1.00, 0.60), III 3.15 (0.85, 1.10, 0.60, 0.60), IV 5.70 (1.60, 2.15, 1.25, 0.70). Habitus like in male, coloration of abdomen slightly paler.

Epigyne (Fig. 4A–C): about 1.1 times wider than long; scape about four times wider than long, strongly rebordered; copulatory openings triangular, located posteriorly; copulatory ducts short, twisted; spermathecae ovoid, touching each other.

##### Variation

. Total length: ♂♂ 2.90–3.60; ♀♀ 6.00–6.50.

##### Distribution

. China (Yunnan).

#### Genus Chorizopesoides

Taxon classificationAnimaliaAraneaeAraneidae

Mi & Wang, 2018

0175E9A5-0187-5CBA-A102-C3E52BF4F809

Chorizopesoides : Mi and Wang 2018: 82

##### Type species

*Chorizopeswulingensis* Yin, Wang and Xie, 1994 from Hunan, China

##### Comments

. The only two species that have been described in this genus were both recorded from China (WSC 2021).

#### Chorizopesoides guoi sp. nov.

Taxon classificationAnimaliaAraneaeAraneidae

4E097BE2-0A25-5EC6-BB80-7B619433C73F

http://zoobank.org/18783528-2199-4DDB-9168-3FED612331CA

Figs 5 , 6 , 20B

##### Type material

**. Holotype.** ♂ (IZCAS-Ar42520), China: Yunnan, Xishuangbanna, Mengla County, Menglun Town, Menglun Nature Reserve, primary tropical seasonal rainforest (21°55.04'N, 101°16.50'E, ca 560 m), 22.VII.2007, G. Zheng leg. ***Paratypes***: 2♂3♀ (IZCAS-Ar42521–42525), same data as holotype; 1♀ (IZCAS-Ar42526), primary tropical seasonal rainforest (21°57.43'N, 101°12.28'E, ca 792 m), 1–15.VI.2007, G. Zheng leg.; 1♂1♀ (IZCAS-Ar42527–42528), secondary tropical seasonal moist forest (21°54.72'N, 101°16.94'E, ca 650 m), 27.VII.2007, G. Zheng leg.; 3♂2♀ (IZCAS-Ar42529–42533), secondary tropical seasonal moist forest (21°54.61'N, 101°17.01'E, ca 630 m), 28.VII.2007, G. Zheng leg.

##### Other material examined

. 1♀ (IZCAS-Ar42534), Xishuangbanna Tropical Botanical Garden, eastern part (21°54.07'N, 101°16.36'E, ca 540 m), 16.VII.2018, X. Mi leg.; 1♀ (IZCAS-Ar42535), Xishuangbanna Tropical Botanical Garden, vine garden (21°55.76'N, 101°15.73'E, ca 490 m), 17.VII.2018, night, X. Mi leg.; 1♀ (IZCAS-Ar42536), G213 roadside near 68 km (21°53.82'N, 101°16.79'E, ca 620 m), 27.VII.2018, X. Mi leg.

##### Comparative material

. *Chorizopesoideswulingensis*, ***Holotype*** ♀, CHINA: Hunan, Sangzhi County, Nanmuping, 17.XIII.1984, J.F. Wang leg. (Fig. 7)

##### Etymology

. The species is named after Professor Guo Zheng, one of the collectors of the type specimens; noun (name) in genitive case.

##### Diagnosis

. The new species resembles *C.wulingensis* and *C.annasestakovae* sp. nov. in appearance, but females can be distinguished from both by the: 1) copulatory ducts spirally coiled vs. circular (Fig. 7C; Mi and Wang, 2018: fig. 3E); 2) pale abdomen with distinct black stripes vs. dark brown abdomen with indistinct stripes; 3) pale cephalon behind eyes vs. dark cephalon with two small, pale spots (Fig. 7D) or without spots (Mi and Wang, 2018: fig. 1C). From *C.wulingensis* by the: 1) scape shorter than half a spermatheca diameter vs. longer than half a spermatheca diameter (Fig. 7); 2) median plate narrower than a spermatheca diameter vs. wider than a spermatheca diameter (Fig. 7); it can be distinguished from *C.annasestakovae* sp. nov. by the: 1) scape not concave vs. concave (Mi and Wang, 2018: fig. 3D–E); 2) shape of the epigyne wider than long vs. as long as wide; 3) palpal tibia wider than long with a cluster of macrosetae vs. as long as tibia width without conspicuous macrosetae (Mi and Wang, 2018: fig. 3A, C); 4) tegulum triangularly elongated (retrolateral view) vs. not so elongated and rounder (Mi and Wang, 2018: fig. 3C); 5) median apophysis slender, two times longer than wide vs. shorter and higher (Mi and Wang, 2018: fig. 3A); 6) tip of the embolus extending beyond the edge of the tegulum (apical view) vs. shorter embolus, not reaching the edge of the tegulum (Mi and Wang, 2018: fig. 3B).

##### Description

**Figure 5.**
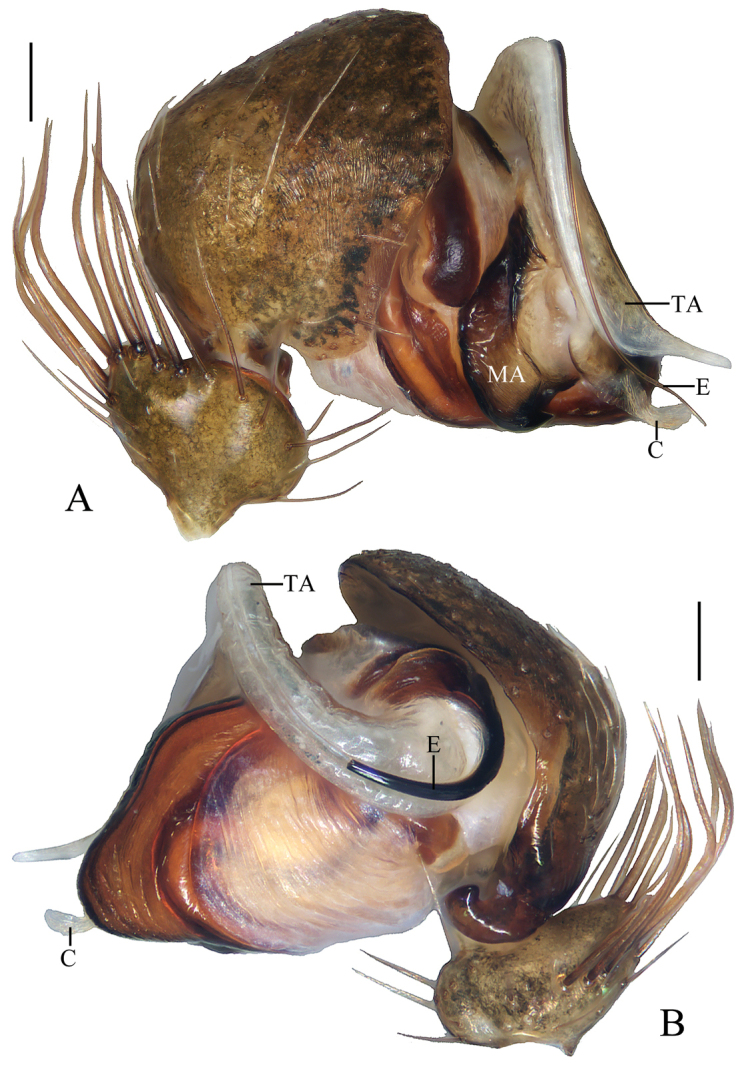
*Chorizopesoidesguoi* sp. nov., holotype, male palp **A** prolateral view **B** retrolateral view (embolus broken). Scale bars: 0.1mm

**Figure 6.**
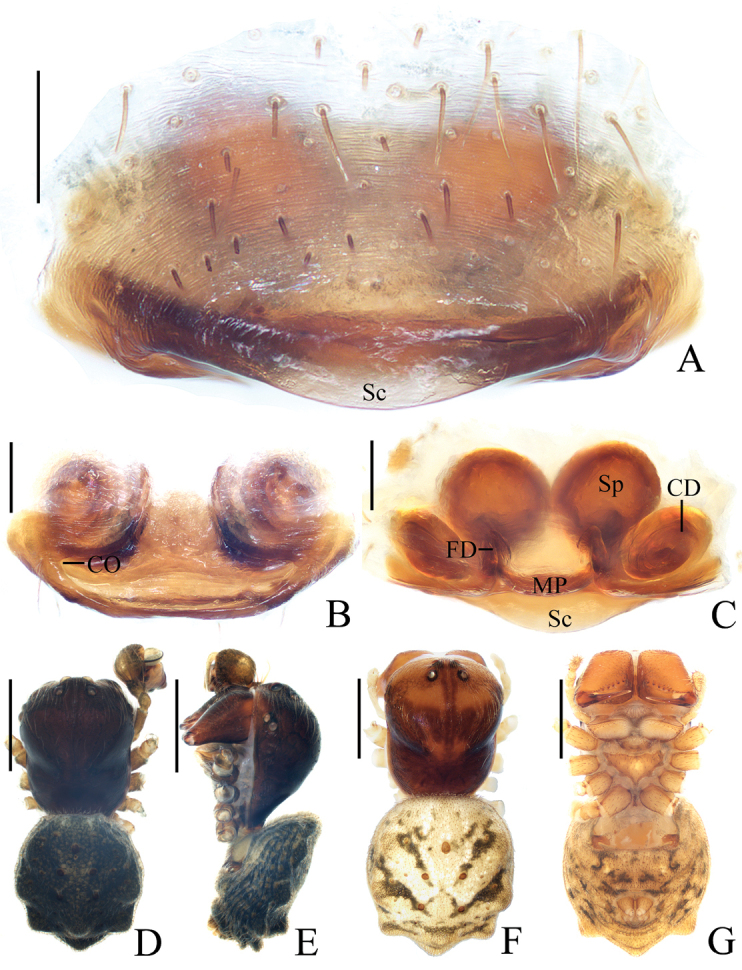
*Chorizopesoidesguoi* sp. nov. **A–C, E, F** female paratype IZCAS-Ar42521 **D, E** holotype **A** epigyne, ventral view **B** ibid., posterior view **C** vulva, dorsal view **D** habitus, dorsal view **E** ibid., lateral view **F** habitus, dorsal view **G** ibid., ventral view. Scale bars: 0.1mm (**A–C**); 1mm (**D–G**)

**. Male** (holotype, Figs 5A, B, 6D, E, 20B). Total length 2.90. Carapace 1.60 long, 0.95 wide. Abdomen 1.65 long, 1.40 wide. Clypeus 0.18 high. Eye sizes and interdistances: AME 0.13, ALE 0.08, PME 0.10, PLE 0.08, AME-AME 0.13, AME-ALE 0.40, PME-PME 0.25, PME-PLE 0.43, MOA length 0.30, anterior width 0.33, posterior width 0.43. Leg measurements: I 3.41 (0.95, 1.13, 0.80, 0.53), II 3.46 (0.90, 1.15, 0.88, 0.53), III 2.40 (0.70, 0.80, 0.50, 0.40), IV 3.29 (0.95, 1.13, 0.73, 0.48). Carapace rectangular, dark brown, with sparse, pale setae, cervical groove obvious. Chelicerae dark brown, five promarginal teeth. Endites wider than long, dark brown basally, paler distally, labium wider than long, triangular, dark brown. Sternum triangular, dark brown, paler medially. Legs yellow with brown annulations. Abdomen about 1.2 times longer than wide, with three pairs of lateral tubercles and three vertical caudal tubercles, dorsum grayish black with lots of brown sigilla; venter grayish black. Spinnerets yellowish brown.

Palp (Figs 5, 20B): tibia with a cluster of macrosetae (approx. 12) distal-dorsally, macrosetae about 1.5 times length of tibia; median apophysis about 1/2 length of bulb diameter in apical view, distal end pointed; embolus extremely long, slender, more than two times length of bulb diameter; terminal apophysis membranous, equal in length to embolus; conductor membranous, shorter than median apophysis in prolateral view.

**Female** (paratype IZCAS-Ar42521, Fig. 6A–C, E, F). Total length 3.55. Carapace 1.75 long, 1.40 wide. Abdomen 2.00 long, 1.75 wide. Clypeus 0.15 high. Eye sizes and interdistances: AME 0.13, ALE 0.08, PME 0.10, PLE 0.08, AME-AME 0.15, AME-ALE 0.45, PME-PME 0.25, PME-PLE 0.48, MOA length 0.30, anterior width 0.30, posterior width 0.40. Leg measurements: I 3.31 (0.90, 1.10, 0.78, 0.53), II 3.19 (0.88, 1.08, 0.75, 0.48), III 2.46 (0.70, 0.83, 0.48, 0.45), IV 3.45 (1.00, 1.25, 0.75, 0.45). Habitus as in male, coloration much paler.

**Figure 7.**
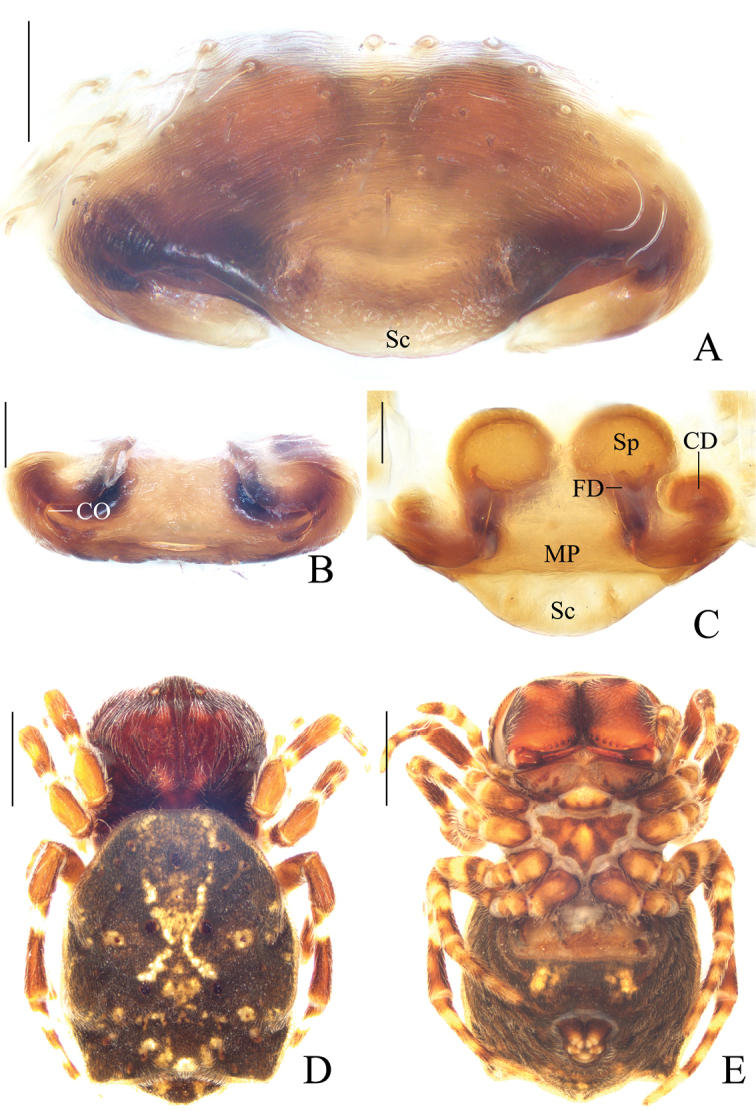
*Chorizopesoideswulingensis*, holotype **A** epigyne, ventral view **B** ibid., posterior view **C** vulva, dorsal view **D** habitus, dorsal view **E** ibid., ventral view. Scale bars: 0.1mm (**A–C**); 1mm (**D, E**)

##### Epigyne

(Fig. 6A–C): about two times wider than long, scape shorter than half a spermatheca diameter; copulatory openings concave, located at lateral side of posterior surface; copulatory ducts long, coiled 720°; spermathecae globular, touching each other.

##### Variation

. Total length: ♂♂ 2.55–3.05; ♀♀ 3.25–4.85.

##### Distribution

. China (Yunnan).

#### Chorizopesoides annasestakovae sp. nov.

Taxon classificationAnimaliaAraneaeAraneidae

C26532FA-A6A9-56C8-BA86-8B93512A7671

http://zoobank.org/DDE89FA3-8775-4EC0-ABDC-05C59F23E69F

Chorizopesoides wulingensis Mi & Wang, 2018: 82, figs 1A–D, 2A–C, 3A–E (misidentified).

##### Type material

**. Holotype.** ♂ (TRU), China: Guizhou, Qiannan, Libo County, Dotang Township, Yaosuo Village, Bizuo (25°16.84'N, 108°4.47'E, ca 601 m), 7–8.VIII.2013, X. Mi & M. Liao leg. (MXQ20130807). ***Paratype*** 1♀(TRU), same data as holotype.

##### Etymology

. The specific name comes from Dr. Anna Šestáková, who confirmed the new species; noun (name) in genitive case.

##### Diagnosis

. See *Chorizopesoidesguoi* sp. nov.

##### Description

. See Mi and Wang (2018).

##### Distribution

. China (Guizhou).

##### Comments

. Compared to the holotype of *C.wulingensis*, the previous description of *C.wulingensis* from Libo County, Guizhou by Mi and Wang (2018) refers to *C.annasestakovae* sp. nov.

#### Genus Deione

Taxon classificationAnimaliaAraneaeAraneidae

Thorell, 1898

A41994C2-6711-5209-9765-95B1B429EA47

Deione Thorell, 1898: 365; DeioneHan et al. 2009: 56; Mi et al. 2010: 35.

##### Type species

*Deionethoracica* Thorell, 1898 from Myanmar

#### Deione cheni sp. nov.

Taxon classificationAnimaliaAraneaeAraneidae

91910149-8AA9-5E2F-96E9-14937CF796B6

http://zoobank.org/13408A71-9FA3-4B2F-BAA6-1919E989F767

Fig. 8

##### Type material

**. Holotype.** ♀ (IZCAS-Ar42537), China: Yunnan, Xishuangbanna, Mengla County, Menglun Town, Menglun Nature Reserve, secondary tropical seasonal moist forest (21°54.72'N, 101°16.94'E, ca 650 m), 16–31.V.2007, G. Zheng leg. ***Paratypes***: 1♀ (IZCAS-Ar42538), Xishuangbanna Tropical Botanical Garden, site 3 around the dump (21°54.34'N, 101°16.79'E, ca 620 m), 2.V.2019, Y. Tong leg.; 2♀ (IZCAS-Ar42539–42540), Xishuangbanna Tropical Botanical Garden, low bamboo plantation (21°53.89'N, 101°16.72'E, ca 570 m), 12.V.2019, Z. Bai leg.

##### Etymology

. The species is named after Mr. Zhigang Chen, one of the collectors of the type specimens; noun (name) in genitive case.

##### Diagnosis

. The new species resembles congeneric species in habitus, but it can be distinguished by the: 1) rhomboid epigyne in ventral view; 2) short, ventrally directed scape.

##### Description

**Figure 8.**
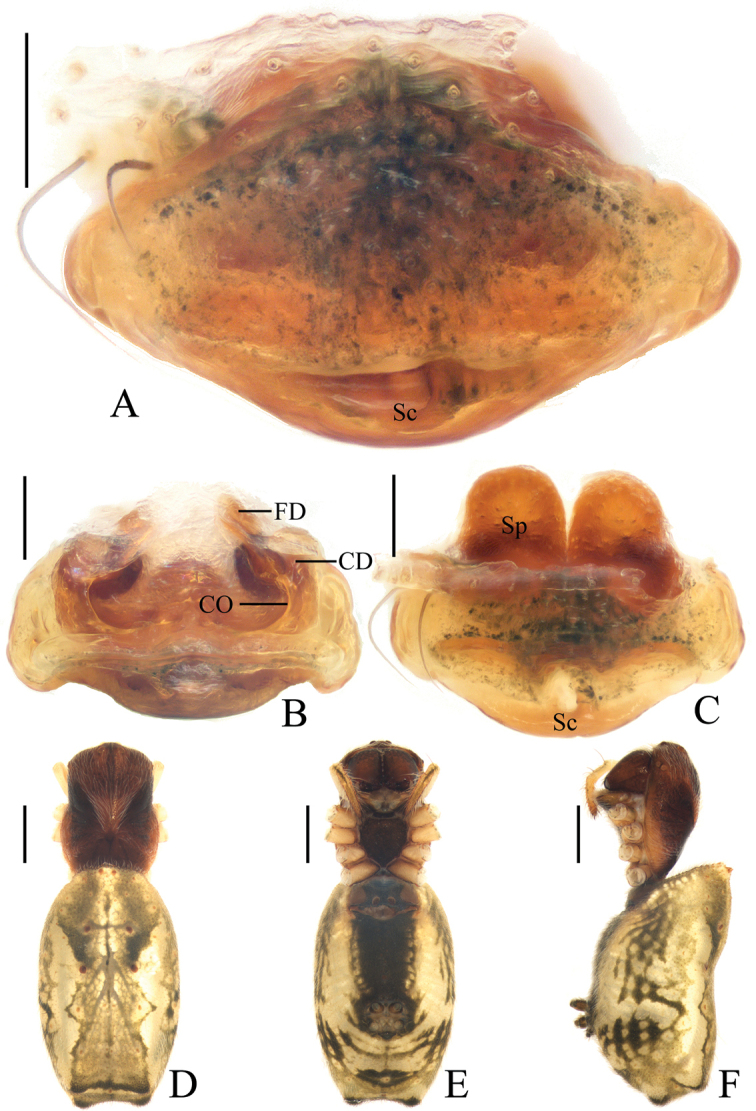
*Deionecheni* sp. nov., holotype **A** epigyne, ventral view **B** ibid., posterior view **C** ibid., anterior view **D** habitus, dorsal view **E** ibid., ventral view **F** ibid., lateral view. Scale bars: 0.1mm (**A–C**); 1mm (**D–F**)

**. Female** (holotype, Fig. 8). Total length 6.30. Carapace 2.50 long, 1.70 wide. Abdomen 4.20 long, 2.50 wide. Clypeus 0.13 high. Eye sizes and interdistances: AME 0.15, ALE 0.10, PME 0.15, PLE 0.10, AME-AME 0.15, AME-ALE 0.53, PME-PME 0.20, PME-PLE 0.58, MOA length 0.43, anterior width 0.35, posterior width 0.45. Leg measurements: I 5.00 (1.50, 1.75, 1.15, 0.60), II 4.95 (1.50, 1.75, 1.15, 0.55), III 3.45 (1.05, 1.25, 0.65, 0.50), IV 4.90 (1.45, 1.80, 1.10, 0.55). Carapace rectangular, dark brown, with dense, pale setae. Chelicerae dark brown, 5 promarginal teeth, 3 retromarginal teeth. Endites dark brown basally, yellow distally, labium triangular, dark brown. Sternum heart-shaped, dark brown. Legs yellow with brown annulations. Abdomen oval, about 1.7 times longer than wide, with two pairs of long setae anteriorly, two pairs of vertically arranged lateral tubercles posteriorly, dorsum yellow with big grayish brown patch, patch with two pairs of constrictions laterally; venter yellow with big grayish black patch medially. Spinnerets grayish yellow, at posterior 1/3 of the abdomen.

Epigyne (Fig. 8A–C): rhomboid, about 1.8 times wider than long, with very short, ventrally directed scape; copulatory openings arcuate; copulatory ducts shorter than a spermatheca length; spermathecae elliptical, touching each other.

##### Variation

. Total length: ♀♀ 4.90–6.30.

##### Distribution

. China (Yunnan).

#### Deione yangi sp. nov.

Taxon classificationAnimaliaAraneaeAraneidae

1E3F9394-18D8-5EDA-9859-EB7DBEC3607B

http://zoobank.org/9FDA3B40-D368-413A-907F-2049B62DD19B

Figs 9 , 10 , 20C

##### Type material

**. Holotype.** ♀ (IZCAS-Ar42541), China: Yunnan, Xishuangbanna, Mengla County, Menglun Town, Menglun Nature Reserve, primary tropical seasonal rainforest (21°57.67'N, 101°11.89'E, ca 790 m), 19–26.IV.2007, G. Zheng leg. ***Paratypes***: 1♀ (IZCAS-Ar42542), primary tropical seasonal rainforest (21°57.59'N, 101°12.21'E, ca 822 m, ca 730 m), 8.VIII.2007, G. Zheng leg.; 1♂ (IZCAS-Ar42543), Xishuangbanna Tropical Botanical Garden, Edible Botanical Garden (21°54.95'N, 101°16.18'E, ca 610 m), 28.VII.2018, X. Mi leg.

##### Comparative material

. *Deionelingulata*, 5♂3♀, China: Hainan, Wuzhishan City, Shuiman Township, Wuzhishan National Natural Reserve (18°54.17'N, 109°41.14'E, ca 870 m), 10.VIII.2020, X. Mi leg. (Figs 11, 20D)

##### Etymology

. The species is named after Mr. Yuanfa Yang (Tongren, Guizhou), one of the collectors of the type specimens; noun (name) in genitive case.

##### Diagnosis

. The new species resembles *D.lingulata* in habitus and copulatory organs but differs in the: 1) thin terminal apophysis, distally the width about equal to the nearest part of embolus vs. terminal apophysis thick, distally about four times wider than the nearest part of the embolus (Han et al. 2009: figs. 12, 13; Fig. 11); 2) median apophysis elliptical in prolateral view vs. triangular (Han et al. 2009: fig. 12; Fig. 11A); 3) scape triangular vs. almost rectangular (Han et al. 2009: figs. 8, 9); and 4) spermathecae touching vs. less than their diameter apart (Han et al. 2009).

**Figure 9.**
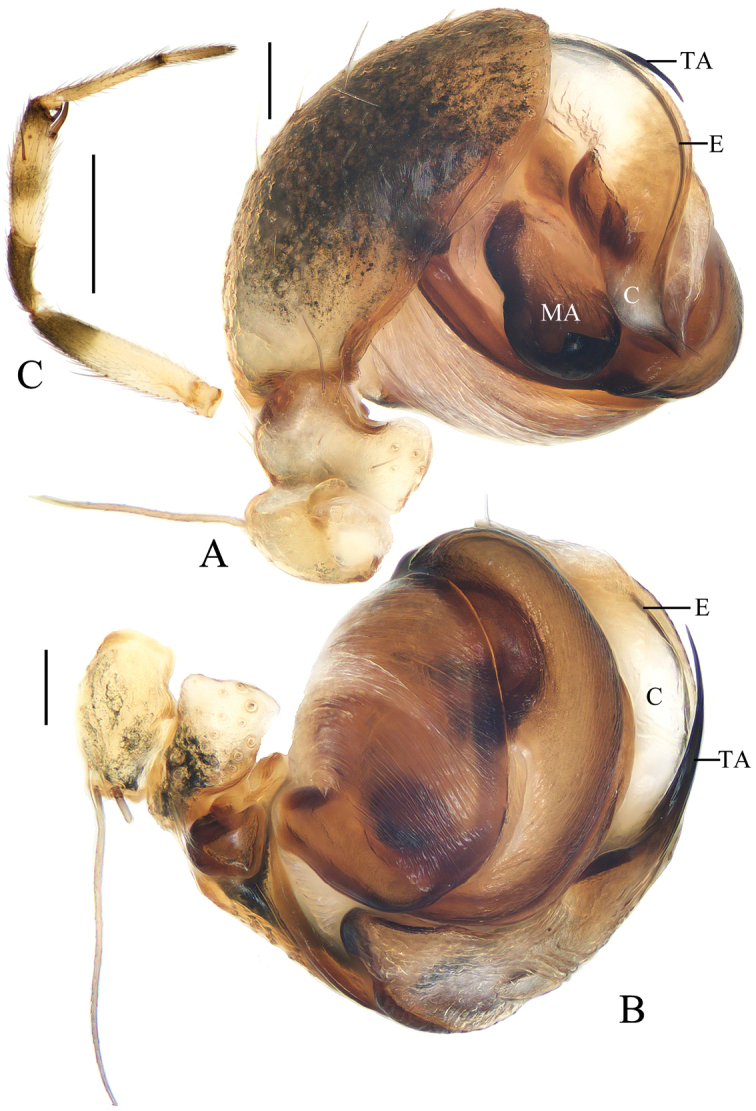
*Deioneyangi* sp. nov., male paratype IZCAS-Ar42543, palp **A** prolateral view **B** retrolateral view **C** left leg II, prolateral view. Scale bars: 0.1mm (**A, B**); 1mm (**C**)

##### Description

**Figure 10.**
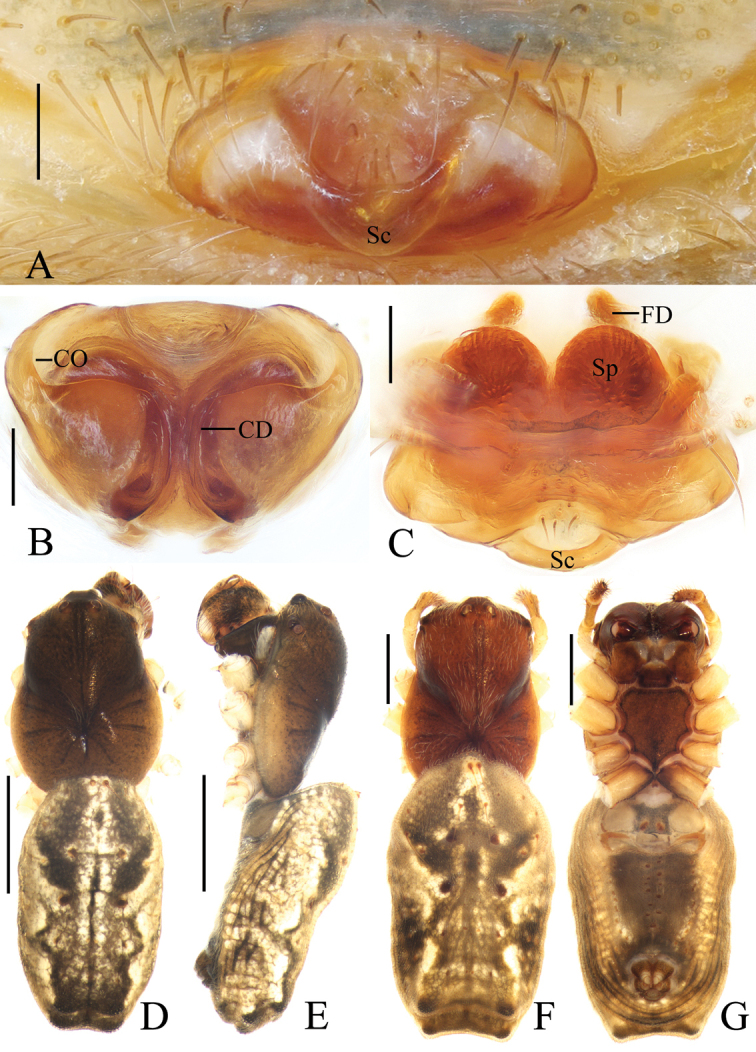
*Deioneyangi* sp. nov. **A–C, F, G** holotype **D, E** male paratype IZCAS-Ar42543 **A** epigyne, ventral view **B** ibid., posterior view **C** vulva, anterior view **D** habitus, dorsal view **E** ibid., lateral view **F** ibid., dorsal view **G** ibid., ventral view. Scale bars: 0.1mm (**A–C**); 1mm (**D–G**)

**. Female** (holotype, Fig. 10A–C, F, G). Total length 6.20. Carapace 2.70 long, 2.00 wide. Abdomen 3.90 long, 2.40 wide. Clypeus 0.10 high. Eye sizes and interdistances: AME 0.18, ALE 0.13, PME 0.15, PLE 0.13, AME-AME 0.15, AME-ALE 0.50, PME-PME 0.20, PME-PLE 0.53, MOA length 0.48, anterior width 0.45, posterior width 0.45. Leg measurements: I 7.70 (2.20, 2.70, 1.90, 0.90), II 6.90 (2.00, 2.40, 1.70, 0.80), III 4.60 (1.50, 1.60, 0.90, 0.60), IV 6.50 (1.90, 2.30, 1.60, 0.70). Carapace almost rectangular, brown, with pale setae, cervical groove obvious. Chelicerae brown, five promarginal teeth, four retromarginal teeth in left chelicera, and five in right. Endites almost rectangular, brown, paler distally, labium triangular, dark brown, paler distally. Sternum heart-shaped, brown. Legs yellow with brown annulations. Abdomen oval, about 1.6 times longer than wide, with two pairs of long setae anteriorly, two pairs of vertically arranged lateral tubercles posteriorly, dorsum grayish brown with yellow patches anteriorly and laterally; venter yellow, big gray patch medially. Spinnerets grayish yellow, at posterior 1/4 of the abdomen.

**Figure 11.**
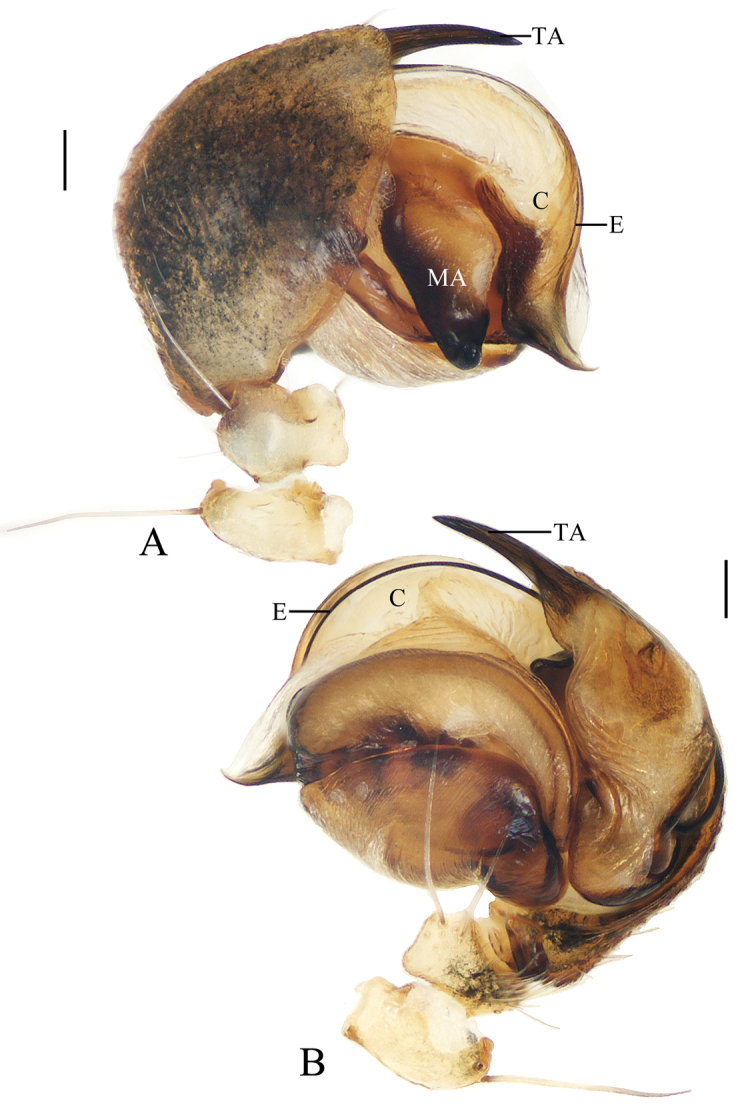
*Deionelingulata*, male palp **A** prolateral view **B** retrolateral view. Scale bars: 0.1mm

Epigyne (Fig. 10A–C): about 1.8 times wider than long; scape triangular; copulatory openings arcuate, situated laterally in posterior view; copulatory ducts longer than spermatheca, arcuate in posterior view; spermathecae globular, touching each other.

**Male** (paratype IZCAS-Ar42543, Figs 9, 10D, E, 20C). Total length 3.75. Carapace 1.70 long, 1.35 wide. Abdomen 2.15 long, 1.30 wide. Clypeus 0.10 high. Eye sizes and interdistances: AME 0.15, ALE 0.10, PME 0.13, PLE 0.10, AME-AME 0.10, AME-ALE 0.23, PME-PME 0.15, PME-PLE 0.28, MOA length 0.38, anterior width 0.35, posterior width 0.38. Leg measurements: I 4.25 (1.25, 1.60, 0.95, 0.45), II 4.35 (1.45, 1.50, 0.95, 0.45), III 2.75 (0.90, 0.95, 0.55, 0.35), IV 3.85 (1.15, 1.45, 0.85, 0.40). Habitus similar to that of female, but tibia II expanded, with 3 macrosetae and chelicerae with 3 retromarginal teeth.

Palp (Figs 9A, B, 20C): median apophysis elliptical in prolateral view, pointed distally; embolus slender, longer than bulb diameter; conductor as wide as bulb, membranous; terminal apophysis spinose, slightly curved.

##### Variation

. Total length: ♀♀ 6.20–6.50.

##### Distribution

. China (Yunnan).

#### Genus Hypsosinga

Taxon classificationAnimaliaAraneaeAraneidae

Ausserer, 1871

552B60BC-6674-5042-9845-FF5618007E47

Hypsosinga Ausserer, 1871: 823; Yin et al. 1997: 306; Yin et al. 2012: 689.

##### Type species.

*Singasanguinea* C.L. Koch, 1844 from Germany

#### Hyposinga pulla sp. nov.

Taxon classificationAnimaliaAraneaeAraneidae

10219213-2841-518F-8B35-F8CBDFB12B2B

http://zoobank.org/6155FE2D-7491-4983-8BFA-A763F57926F4

Figs 12 , 13 , 21A

##### Type material

**. *Holotype*** ♂ (IZCAS-Ar42544), China: Yunnan, Xishuangbanna, Mengla County, Menglun Town, Menglun Nature Reserve, G213 roadside, Mannanxing (21°53.49'N, 101°17.11'E, ca 560 m), 9.VIII.2018, C. Wang leg. ***Paratypes***: 1♀ (IZCAS-Ar42545), same data as holotype; 2♂ (IZCAS-Ar42546–42547), along G213 roadside (21°53.55'N, 101°16.39'E, ca 540 m), 3.VIII.2018, C. Wang leg.

##### Etymology

. The specific name comes from the Latin word “pulla”, meaning “dark, blackish”, referring to the dark markings at the eye region; adjective.

##### Diagnosis

. The new species resembles *H.pygmaea* (Sundevall, 1831) in habitus but can be distinguished by the: 1) enlarged copulatory ducts vs. not enlarged (Yin et al. 1997: fig. 215d); 2) spermathecae touching vs. separated from each other (Yin et al. 1997: fig. 215d); 3) embolus length less than half a bulb diameter vs. longer than a bulb diameter (Yin et al. 1997: fig. 215e).

##### Description

**Figure 12.**
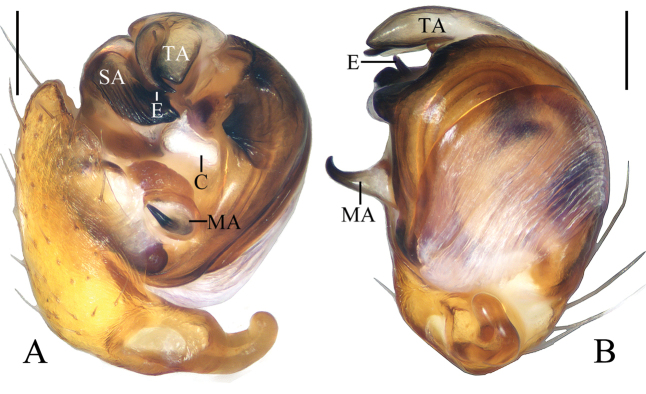
*Hyposingapulla* sp. nov., holotype, male palp **A** prolateral view **B** ventral view. Scale bars: 0.1mm

**Figure 13.**
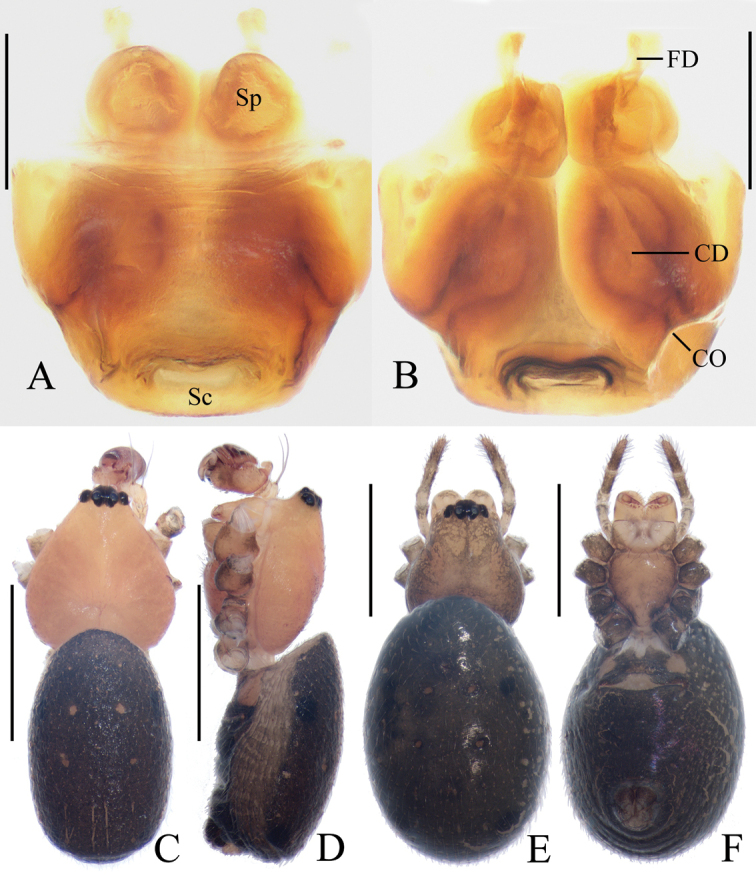
*Hyposingapulla* sp. nov. **A, B, E, F** female paratype IZCAS-Ar42545 **C, D** holotype **A** epigyne, anterior view **B** ibid., posterior view **C** habitus, dorsal view **D** ibid., lateral view **E** ibid., dorsal view **F** ibid., ventral view. Scale bars: 0.1mm (**A, B**); 1mm (**C–F**)

**. Male** (holotype, Figs 12, 13C, D, 21A). Total length 2.40. Carapace 1.10 long, 0.95 wide. Abdomen 1.45 long, 0.90 wide. Clypeus 0.18 high. Eye sizes and interdistances: AME 0.06, ALE 0.04, PME 0.05, PLE 0.04, AME-AME 0.05, AME-ALE 0.03, PME-PME 0.05, PME-PLE 0.05, MOA length 0.15, anterior width 0.15, posterior width 0.15. Leg measurements: I 4.00 (1.30, 1.35, 0.90, 0.45), II 3.60 (1.15, 1.20, 0.85, 0.40), III 2.45 (0.80, 0.75, 0.55, 0.35), IV 3.75 (1.25, 1.20, 0.90, 0.40). Carapace pear shaped, yellow with black patch in eye region, cervical groove inconspicuous, fovea transverse. Chelicerae yellow, three promarginal teeth, two retromarginal teeth. Endites yellow, labium triangular, yellow. Sternum yellow, with sparse, dark setae. Legs grayish brown, without annulations. Abdomen elliptical, about 1.6 times longer than wide, covered with pale setae, dorsum black-brown; venter grayish brown with big black patch medially. Spinnerets brownish black.

Palp (Figs 12, 21A): with two patellar bristles; median apophysis hooked; embolus shorter than half bulb diameter, covered by terminal and subterminal apophyses in prolateral view; conductor thick; terminal apophysis membranous, bifurcated distally; subterminal apophysis membranous, width almost same as width of terminal apophysis; tegulum extended near conductor.

**Female** (paratype IZCAS-Ar42545, Fig. 13A, B, E, F). Total length 2.75. Carapace 1.00 long, 0.90 wide. Abdomen 2.00 long, 1.45 wide. Clypeus 0.13 high. Eye sizes and interdistances: AME 0.06, ALE 0.04, PME 0.05, PLE 0.04, AME-AME 0.05, AME-ALE 0.05, PME-PME 0.05, PME-PLE 0.05, MOA length 0.15, anterior width 0.15, posterior width 0.18. Leg measurements: I 3.10 (0.95, 1.05, 0.70, 0.40), II 2.85 (0.90, 0.95, 0.65, 0.35), III 2.00 (0.65, 0.60, 0.45, 0.30), IV 3.00 (1.00, 0.95, 0.70, 0.35). Habitus similar to that of male; carapace a little darker.

Epigyne (Fig. 13A, B): pentagonal in anterior view, about 1.4 times wider than long; copulatory openings laterally situated; copulatory ducts large, wider than spermatheca diameter at its widest part; spermathecae globular, touching each other.

##### Variation

. Total length: ♂♂ 2.40–2.55.

##### Distribution

. China (Yunnan).

#### Genus Mangora

Taxon classificationAnimaliaAraneaeAraneidae

O. Pickard-Cambridge, 1889

258AADFD-DA82-56EC-9812-DE24C1F3D14B

Mangora O. Pickard-Cambridge, 1889: 14; Yin et al. 1997: 329; Yin et al. 2012: 711.

##### Type species

*Mangorapicta* O. Pickard-Cambridge, 1889 from Guatemala

##### Comments

. Unlike the typical *Mangora* species, the two new species in this region both lack trichobothria on the tibia of leg III, but they have some common characters with *Mangora*, such as the cephalic region of the carapace is about half the maximum width of the thoracic region, the palp with one patellar bristle, and the abdomen oval; thus, we place the two species in *Mangora*, and phylogenetic analysis will focus on the placement of the two new species.

#### Mangora baii sp. nov.

Taxon classificationAnimaliaAraneaeAraneidae

75A2D150-ECD7-5A86-9EF3-BEA82A8B8978

http://zoobank.org/9A48184E-E930-4177-B798-24A4B8A7C89D

Figs 14 , 15 , 21B

##### Type material

**. Holotype.** ♂ (IZCAS-Ar42548) China: Yunnan, Xishuangbanna, Mengla County, Menglun Town, Menglun Nature Reserve, secondary forest near mountain top (21°57.92'N, 101°12.05'E, ca 820 m), 2.VI.2013, Z. Zhao & Z. Chen leg. ***Paratypes***: 1♀ (IZCAS-Ar42549), secondary tropical seasonal rainforest (21°55.43'N, 101°16.44'E, ca 600 m), 19–25.XI.2006, G. Zheng leg.; 1♀ (IZCAS-Ar42550), secondary tropical seasonal moist forest (21°54.72'N, 101°16.94'E, ca 650 m), 19–25.XI.2006, G. Zheng leg.; 1♀ (IZCAS-Ar42551), secondary tropical seasonal rainforest (21°55.43'N, 101°16.44'E, ca 600 m), 5–12.XII.2006, G. Zheng leg.; 1♀ (IZCAS-Ar42552), secondary tropical seasonal rainforest (21°55.43'N, 101°16.44'E, ca 600 m), 19–26.V.2007, G. Zheng leg.; 1 ♂ (IZCAS-Ar42638), G213 roadside, secondary forest (21°54.46'N, 101°16.76'E, ca 640 m), 20.XI.2009, G. Tang leg.; 1♂(IZCAS-Ar42562), secondary forest near mountain top (21°57.96'N, 101°12.19'E, ca 787 m), 31.V.2013, Z. Zhao & Z. Chen leg.; 1♂ (IZCAS-Ar42639), secondary tropical forest, around garbage dump (21°54.17'N, 101°16.87'E, ca 609 m), 31.VII.2018, Z. Bai leg.

##### Etymology

. The species is named after Mr. Zilong Bai, one of the collectors of the type specimens; noun (name) in genitive case.

##### Diagnosis

. The new species differs from congeners by the following combination of characters: 1) the abdomen has two transverse patches and one longitudinal patch; 2) the scape is distally widened; 3) the conductor is long, membranous, and basally trifurcated.

##### Description

**Figure 14.**
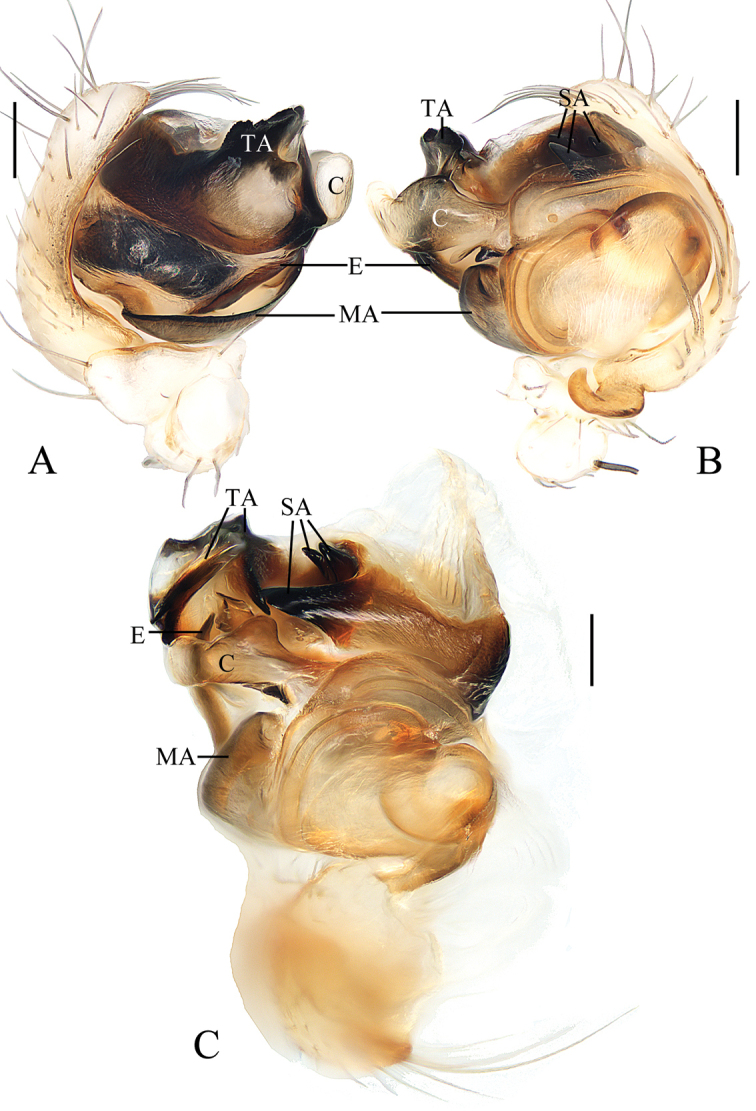
*Mangorabaii* sp. nov., male palp **A, B** holotype **C** male paratype IZCAS-Ar42638 **A** prolateral view **B** ventral view **C** expanded in lactic acid. Scale bars: 0.1mm

**Figure 15.**
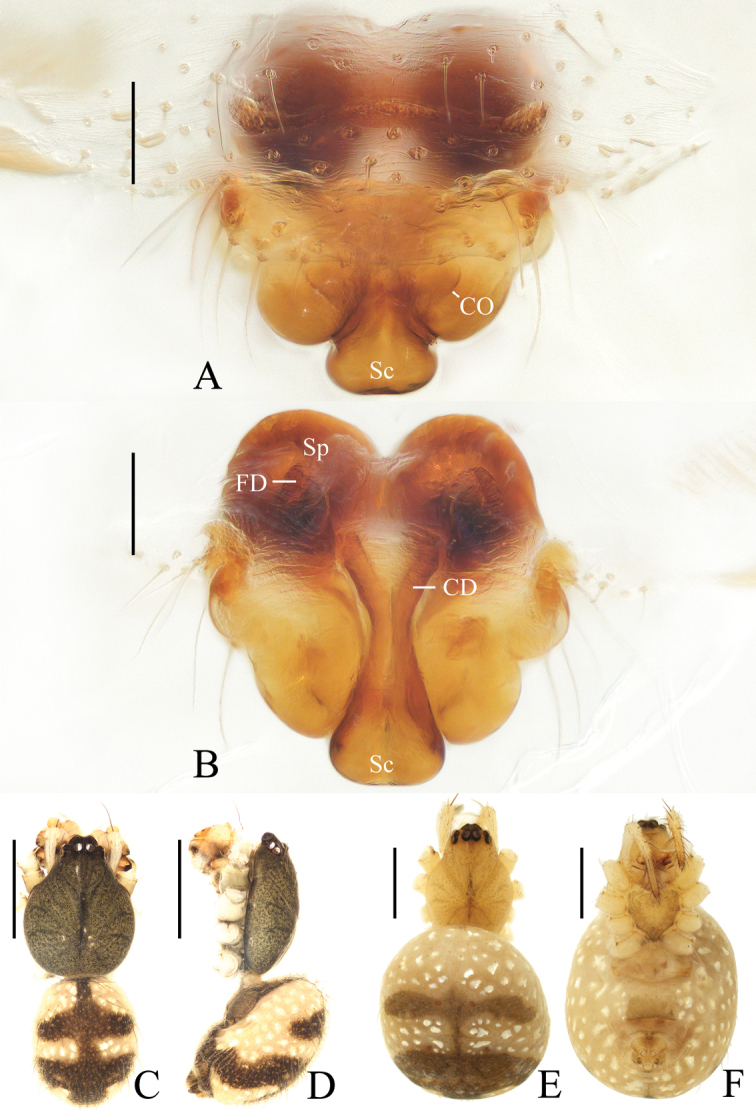
*Mangorabaii* sp. nov. **A, B, E, F** female paratype IZCAS-Ar42551 **C, D** holotype **A** epigyne, ventral view **B** ibid., posterior view **C** habitus, dorsal view **D** ibid., lateral view **E** ibid., dorsal view **F** ibid., ventral view. Scale bars: 0.1mm (**A, B**); 1mm (**C–F**)

**. Male** (holotype, Figs 14A, B, 15C, D, 21B). Total length 2.60. Carapace 1.40 long, 1.10 wide. Abdomen 1.55 long, 1.00 wide. Clypeus 0.08 high. Eye sizes and interdistances: AME 0.10, ALE 0.05, PME 0.08, PLE 0.05, AME-AME 0.08, AME-ALE 0.03, PME-PME 0.03, PME-PLE 0.10, MOA length 0.30, anterior width 0.30, posterior width 0.20. Leg measurements: I 5.00 (1.35, 1.75, 1.30, 0.60), II 4.40 (1.30, 1.40, 1.15, 0.55), III 2.95 (0.90, 0.95, 0.70, 0.40), IV 4.20 (1.30, 1.35, 1.05, 0.50). Carapace pear shaped, grayish yellow with black eye region, cervical groove slightly obvious, fovea longitudinal. Chelicerae yellow, four promarginal and three retromarginal teeth. Endites grayish yellow, with a protuberance on anterior lateral margin, labium grayish brown, paler distally. Sternum heart-shaped, grayish yellow. Legs yellow without annulations, femur II with furrow basally, tibia II with 13 macrosetae. Abdomen oval, about 1.55 times longer than wide, with long, grayish brown setae, dorsum grayish yellow with two transverse and one longitudinal grayish brown patch; venter grayish yellow, big grayish brown patch medially. Spinnerets grayish yellow.

Palp (Figs 14, 21B): with one patellar bristle; cymbium tip with cluster of long macrosetae, median apophysis about a bulb diameter width, lamellar; embolus tapered and slightly curved; conductor length about equal to bulb diameter, membranous, basally trifurcated; terminal apophysis extremely large, almost triangular in apical view; subterminal apophysis with 3 protuberances.

**Female** (paratype IZCAS-Ar42551, Fig. 15A, B, E, F). Total length 3.85. Carapace 1.75 long, 1.20 wide. Abdomen 2.65 long, 2.30 wide. Clypeus 0.05 high. Eye sizes and interdistances: AME 0.13, ALE 0.10, PME 0.13, PLE 0.10, AME-AME 0.08, AME-ALE 0.05, PME-PME 0.03, PME-PLE 0.10, MOA length 0.35, anterior width 0.30, posterior width 0.25. Leg measurements: I 6.10 (1.75, 2.10, 1.55, 0.70), II 5.15 (1.50, 1.75, 1.25, 0.65), III 3.50 (1.10, 1.15, 0.75, 0.50), IV 5.15 (1.55, 1.75, 1.25, 0.60). Habitus similar to that of male but endites without protuberances.

Epigyne (Fig. 15A, B): wider than long, with distally widened scape; copulatory openings narrow, situated at anterior base of lateral lobes; copulatory ducts slightly curved; spermathecae globular, touching each other.

##### Variation

. Total length: ♂♂ 2.45–2.60; ♀♀ 3.10–3.85.

##### Distribution

. China (Yunnan).

#### Mangora cephala sp. nov.

Taxon classificationAnimaliaAraneaeAraneidae

1F7D6EF7-FE9E-53A3-8F1A-1CA0DE146D88

http://zoobank.org/580FDD3B-F09F-48B5-BB94-A59A9A8B6483

Figs 16 , 17 , 21C

##### Type material

**. Holotype.** ♂ (IZCAS-Ar42553), China: Yunnan, Xishuangbanna, Mengla County, Menglun Town, Menglun Nature Reserve, secondary tropical montane evergreen broad-leaf forest (21°57.53'N, 101°12.30'E, ca 860 m), 19–25.XI.2006, G. Zheng leg. ***Paratypes***: 1♀ (IZCAS-Ar42554), rubber plantation (approx. 20 years old) (21°54.65'N, 101°16.26'E, ca 570 m), 16–24.IX.2006, G. Zheng leg.; 1♂ (IZCAS-Ar42555), rubber plantation (approx. 20 years old) (21°54.67'N, 101°16.26'E, ca 570 m), 5–12.X.2006, G. Zheng leg.; 1♀ (IZCAS-Ar42556), rubber plantation (approx. 20 years old) (21°54.46'N, 101°15.98'E, ca 570 m), 5–12.X.2006, G. Zheng leg.; 1♀ (IZCAS-Ar42557), rubber plantation (approx. 20 years old) (21°54.68'N, 101°16.32'E, ca 590 m), 5–12.X.2006, G. Zheng leg.; 1♀ (IZCAS-Ar42558), rubber plantation (approx. 20 years old) (21°54.46'N, 101°15.98'E, ca 570 m), 19–25.X.2006, G. Zheng leg.; 1♀ (IZCAS-Ar42559), rubber plantation (approx. 20 years old) (21°54.67'N, 101°16.26'E, ca 570 m), 19–26.V.2007, G. Zheng leg.; 1♂ (IZCAS-Ar42560), rubber plantation (approx. 20 years old) (21°54.463'N, 101°15.978'E, 569 m), 5–12.XII.2006, G. Zheng leg.; 1♂ (IZCAS-Ar42561), *Paramicheliabaillonii* plantation (approx. 20 years old) (21°54.77'N, 101°16.04'E, ca 560 m), 19–25.XII.2006, G. Zheng leg.

##### Etymology

. The specific name is derived from the Greek word “cephalos”, meaning “head”, referring to the brown cephalic region of the females; noun in apposition.

##### Diagnosis

. The new species differs from congeneric species by the following combination of characters: 1) a somewhat rectangular scape; 2) a slender, translucent subterminal apophysis; 3) a tegular protuberance near the base of the median apophysis; and 4) an abdomen with an arcuate brown patch anteriorly and four transverse brown patches medially and posteriorly.

##### Description

**Figure 16.**
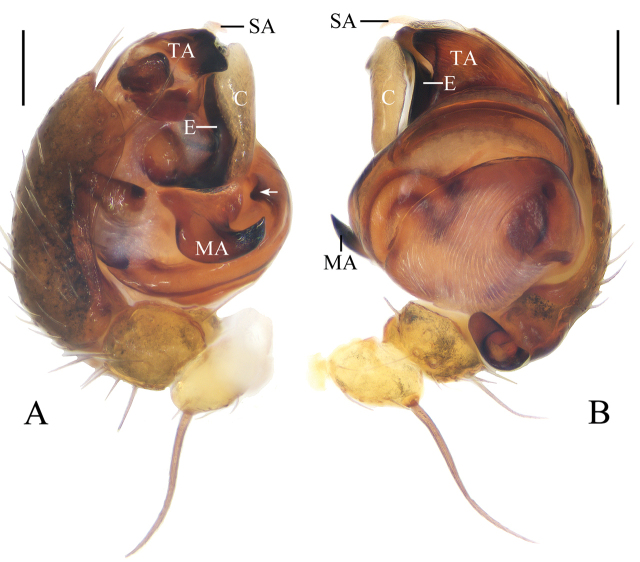
*Mangoracephala* sp. nov., holotype, male palp **A** prolateral view **B** retrolateral view. Scale bars: 0.1mm

**Figure 17.**
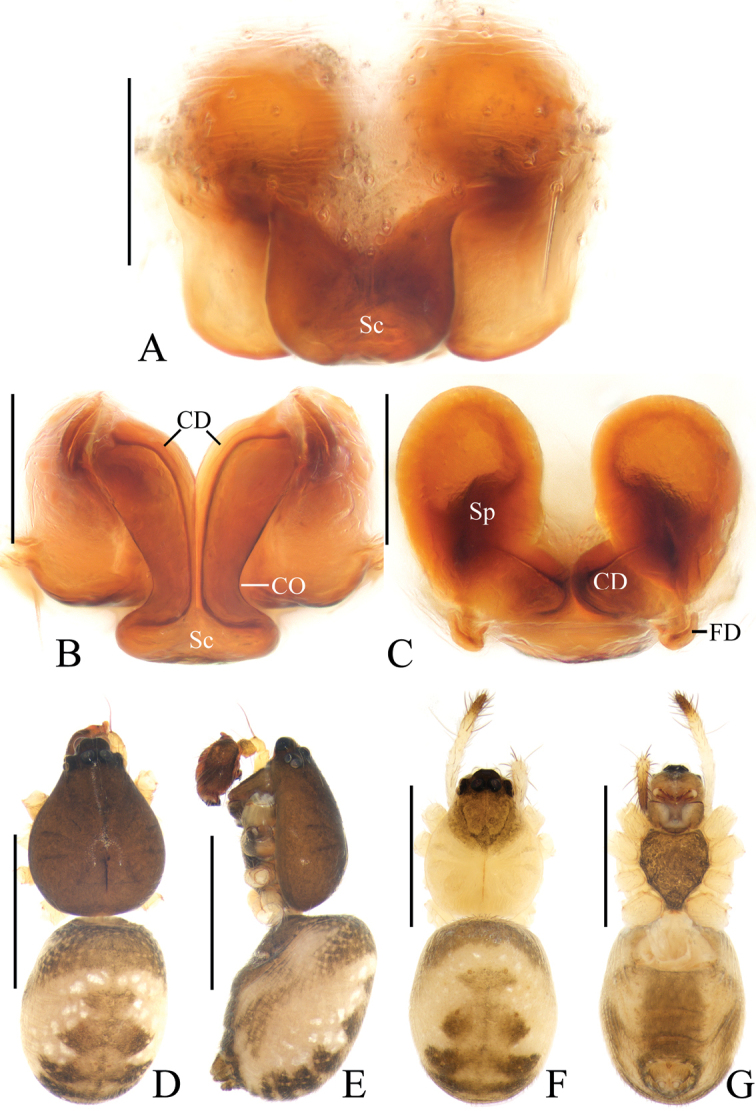
*Mangoracephala* sp. nov. **A–C, F, G** female paratype IZCAS-Ar42559, **D, E** holotype **A** epigyne, ventral view **B** ibid., posterior view **C** vulva, dorsal view **D** habitus, dorsal view **E** ibid., lateral view **F** ibid., dorsal view **G** ibid., ventral view. Scale bars: 0.1mm (**A–C**); 1mm (**D–G**)

**. Male** (holotype, Figs 16, 17D, E, 21C). Total length 2.40. Carapace 1.20 long, 0.90 wide. Abdomen 1.20 long, 1.00 wide. Clypeus 0.10 high. Eye sizes and interdistances: AME 0.10, ALE 0.05, PME 0.08, PLE 0.05, AME-AME 0.08, AME-ALE 0.05, PME-PME 0.03, PME-PLE 0.08, MOA length 0.23, anterior width 0.25, posterior width 0.20. Leg measurements: I 3.90 (1.20, 1.25, 1.00, 0.45), II 3.30 (1.00, 1.00, 0.90, 0.40), III 2.20 (0.70, 0.70, 0.50, 0.30), IV 3.40 (1.05, 1.05, 0.90, 0.40). Carapace pear shaped, dark brown, cervical groove inconspicuous, fovea longitudinal. Chelicerae dark brown, four promarginal teeth, two retromarginal teeth. Endites and labium dark brown, paler distally. Sternum dark brown with indistinct, darker radial patches. Legs yellow without annulations, femur II with a furrow basally, tibia II with seven macrosetae. Abdomen elliptical, about 1.2 times longer than wide, dorsum yellow with arcuate brown patch anteriorly, four transverse brown patches medially and posteriorly; venter grayish yellow laterally, brown medially. Spinnerets grayish yellow.

Palp (Figs 16, 21C): with one patellar bristle; tegulum with a protuberance near base of median apophysis (see arrow in Fig. 16A); median apophysis hooked; embolus thick, slightly curved; conductor membranous, as long as embolus in prolateral view; terminal apophysis prominent, pointed distally; subterminal apophysis slender, translucent.

**Female** (paratype IZCAS-Ar42559, Figs 17A–C, F, G). Total length 2.45. Carapace 1.10 long, 0.85 wide. Abdomen 1.60 long, 1.10 wide. Clypeus 0.03 high. Eye sizes and interdistances: AME 0.10, ALE 0.05, PME 0.08, PLE 0.05, AME-AME 0.08, AME-ALE 0.05, PME-PME 0.05, PME-PLE 0.08, MOA length 0.23, anterior width 0.23, posterior width 0.20. Leg measurements: I 4.10 (1.25, 1.30, 1.05, 0.50), II 3.65 (1.15, 1.20, 0.90, 0.40), III 2.30 (0.75, 0.70, 0.50, 0.35), IV 3.50 (1.05, 1.10, 0.90, 0.45). Habitus similar to that of male but thoracic region yellow.

Epigyne (Fig. 17A–C) about 1.1 times wider than long; with rectangular scape; copulatory openings wide, covered by lateral part of scape in ventral view; copulatory ducts long, slightly curved, covered by posterior plate in posterior view; spermathecae ovoid, separated from each other.

##### Variation

. Total length: ♂♂ 2.10–2.40; ♀♀ 2.10–2.60.

##### Distribution

. China (Yunnan).

#### Genus Milonia

Taxon classificationAnimaliaAraneaeAraneidae

Thorell, 1890

CECA6888-9A46-56DF-934C-52378D074DB1

Milonia Thorell, 1890: 180.

##### Type species

*Miloniabrevipes* Thorell, 1890 from Sumatra

##### Comments

. This is a poorly understood genus; all seven species were described more than 100 years ago. Among them, two are known from juveniles, five are known from a single-sex, and no high-quality illustrations of the genitalia were provided in the published literature. We place the new species in this genus based on the following characters: large chelicerae, cylindrical abdomen in female, relatively stout legs, spinnerets of the female situated at the middle part of the ventral abdomen.

#### Milonia gemella sp. nov.

Taxon classificationAnimaliaAraneaeAraneidae

D2C43CAF-15D0-574F-9B61-EBD0F3CEB04C

http://zoobank.org/E7B60026-AE2F-4931-9A75-F1FA4E6E23EA

Figs 18 , 19 , 21D

##### Type material

**. Holotype.** ♂ (IZCAS-Ar42562), China: Yunnan, Xishuangbanna, Mengla County, Menglun Town, Menglun Nature Reserve, G213 roadside, secondary forest (21°54.46'N, 101°16.76'E, ca 640 m), 20.XI.2009, G. Tang leg. ***Paratypes***: 1♀ (IZCAS-Ar42563), primary tropical seasonal rainforest (21°57.53'N, 101°12.38'E, ca 899 m), 4–11.V.2007, G. Zheng leg.; 1♀ (IZCAS-Ar42564), secondary tropical forest, around garbage dump (21°54.17'N, 101°16.87'E, ca 609 m), 31.VII.2018, Z. Bai leg.; 1♀ (IZCAS-Ar42565), Xishuangbanna Tropical Botanical Garden, site 1 around the dump (21°54.28'N, 101°16.75'E, ca 630 m), 25.IV.2019, Z. Bai leg.

##### Etymology

. The specific name is from the Latin word “gemella”, meaning “twin born”, referring to the two white spots on the abdomen ventrally; adjective.

##### Diagnosis

. The new species can be distinguished from congeneric species by the following combination of characters: 1) dorsal abdomen with two pairs of small grayish brown spots medially and a large dark brown spot posteriorly; 2) triangular scape; and 3) prominent and bifurcated terminal apophysis.

##### Description

**Figure 18.**
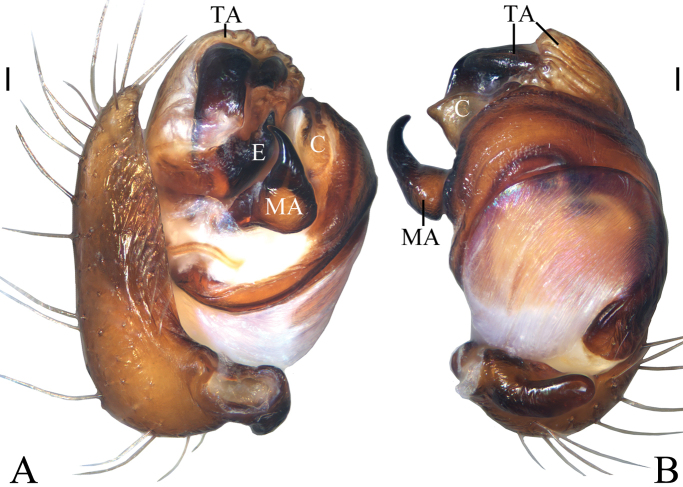
*Miloniagemella* sp. nov., holotype, male palp **A** prolateral view **B** ventral view. Scale bars: 0.1mm

**Figure 19.**
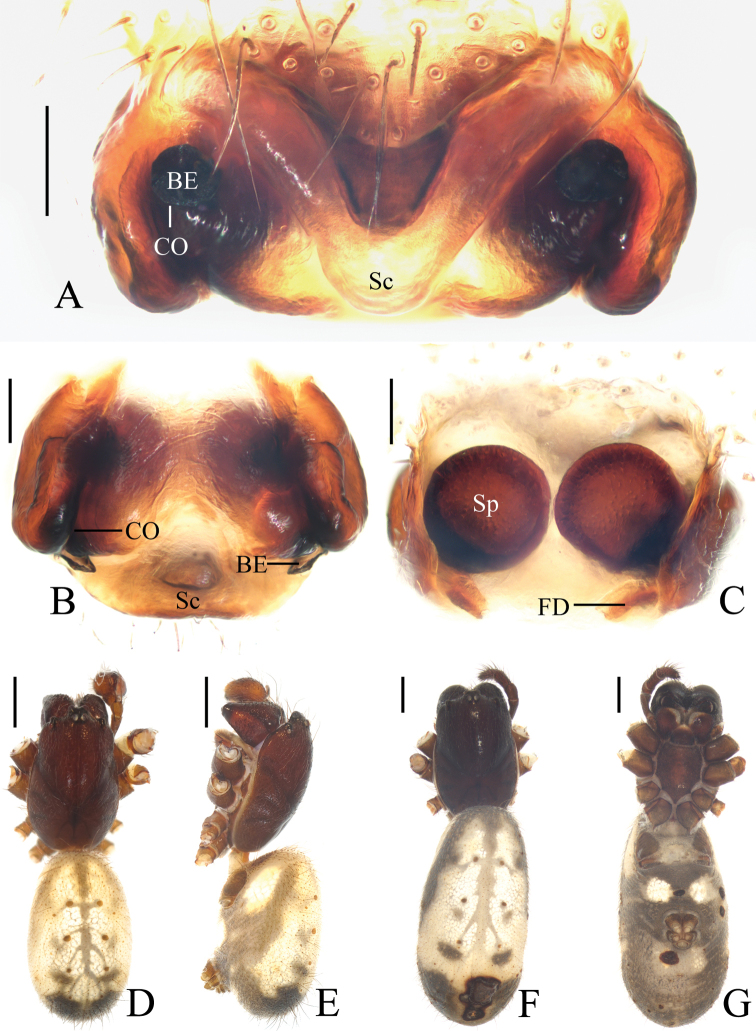
*Miloniagemella* sp. nov. **A–C, F, G** female paratype IZCAS-Ar42564 **D, E** holotype **A** epigyne, ventral view **B** ibid., posterior view **C** vulva, dorsal view **D** habitus, dorsal view **E** ibid., lateral view **F** ibid., dorsal view **G** ibid., ventral view. Scale bars: 0.1 mm (**A–C**); 1mm (**D–G**)

**. Male** (holotype, Figs 18, 19D, E, 21D). Total length 6.30. Carapace 2.90 long, 1.80 wide. Abdomen 3.40 long, 2.10 wide. Clypeus 0.13 high. Eye sizes and interdistances: AME 0.15, ALE 0.10, PME 0.13, PLE 0.10, AME-AME 0.23, AME-ALE 0.28, PME-PME 0.05, PME-PLE 0.38, MOA length 0.38, anterior width 0.40, posterior width 0.28. Leg measurements: I 7.30 (2.10, 2.75, 1.55, 0.90), II 6.75 (1.90, 2.40, 1.60, 0.85), III 4.00 (1.25, 1.40, 0.75, 0.60), IV 5.30 (1.65, 2.00, 1.05, 0.60). Carapace elliptical, brown, with pale setae, cervical groove obvious. Chelicerae brown, four promarginal teeth, three retromarginal teeth. Endites brown, paler distally, labium triangular, brown, paler distally. Sternum pentagonal, dark brown with pale setae. Legs brown, without annulations. Abdomen elliptical, about 1.6 times longer than wide, covered with dark setae, dorsum yellow with two pairs of grayish brown spots medially, big black spot posteriorly; venter yellowish brown with pair of white spots. Spinnerets yellowish brown, at posterior 1/3 of the abdomen.

Palp (Figs 18, 21D): with two patellar bristles; median apophysis stout at base, with a slender, curved spur; embolus broad at base, abruptly tapered to a fine tip; conductor broad at base, tapering to a narrow tip; terminal apophysis extremely large, strongly sclerotized, bifurcated distally, one long, narrow branch, one shorter, wider branch (see arrows in Fig. 21D).

**Female** (paratype IZCAS-Ar42564, Fig. 19A–C, F, G). Total length 9.40. Carapace 3.60 long, 2.40 wide. Abdomen 6.10 long, 3.10 wide. Clypeus 0.23 high. Eye sizes and interdistances: AME 0.18, ALE 0.10, PME 0.13, PLE 0.10, AME-AME 0.15, AME-ALE 0.45, PME-PME 0.05, PME-PLE 0.63, MOA length 0.45, anterior width 0.45, posterior width 0.28. Leg measurements: I 8.45 (2.40, 3.05, 2.05, 0.95), II 7.55 (2.10, 2.85, 1.75, 0.85), III 4.20 (1.00, 1.75, 0.85, 0.60), IV 6.40 (1.95, 2.50, 1.25, 0.70). Habitus similar to that of male but abdomen about two times longer than wide.

Epigyne (Fig. 19A–C) about two times wider than long; short, triangular scape, flanked by round copulatory openings; copulatory ducts shorter than a spermatheca diameter; spermathecae globular, touching each other.

**Figure 20.**
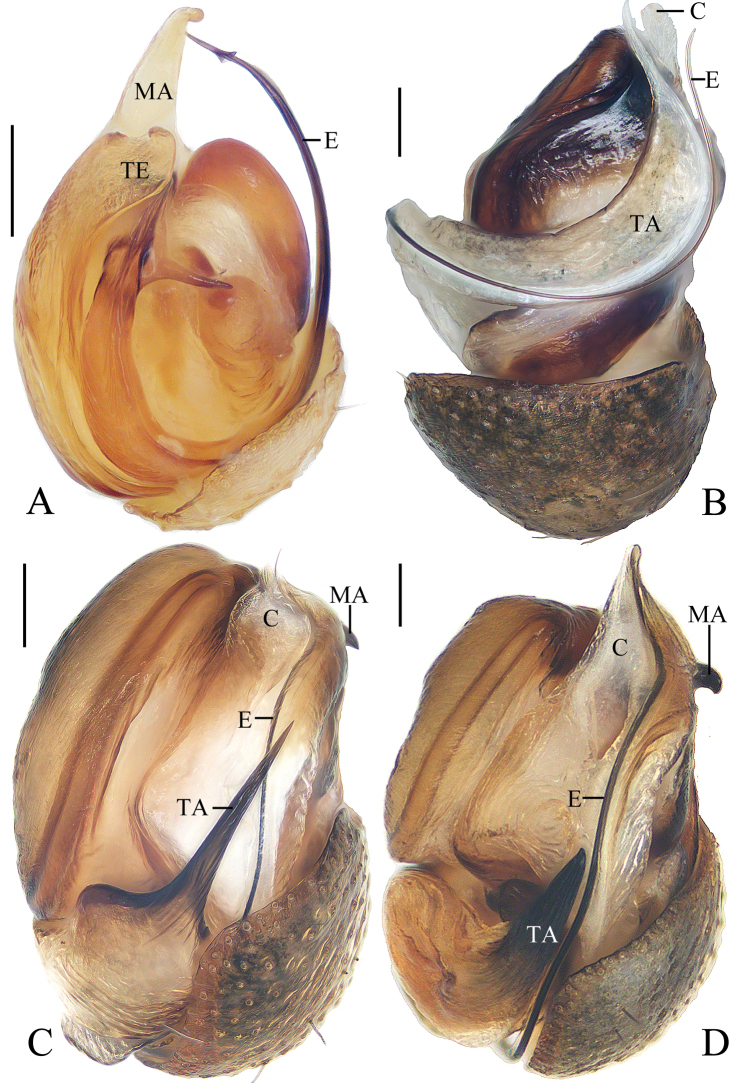
Male palps, apical view **A***Acusilastongi* sp. nov. **B***Chorizopesoidesguoi* sp. nov. **C***Deioneyangi* sp. nov. **D***Deionelingulata*. Scale bars: 0.1

##### Variation

. Total length: ♀♀ 9.20–9.40.

**Figure 21.**
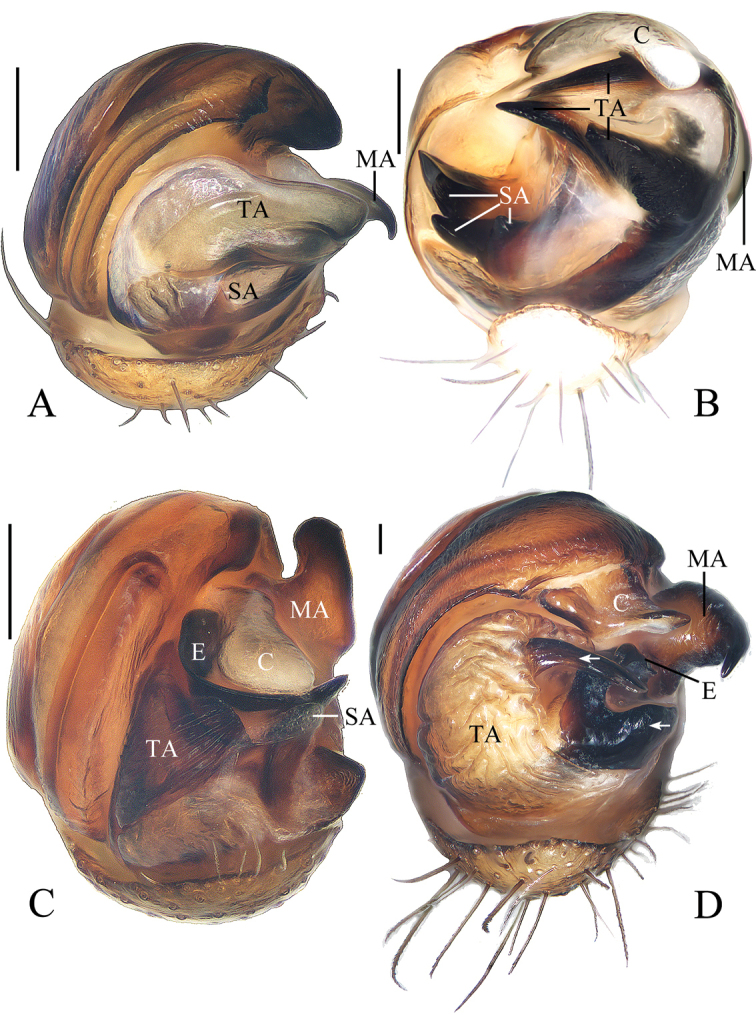
Male palps, apical view **A***Hyposingapulla* sp. nov. **B***Mangorabaii* sp. nov. **C***Mangoracephala* sp. nov. **D***Miloniagemella* sp. nov. Scale bars: 0.1mm

##### Distribution

. China (Yunnan).

## Supplementary Material

XML Treatment for Acusilas

XML Treatment for Acusilas tongi

XML Treatment for Chorizopes

XML Treatment for Chorizopes yui

XML Treatment for Chorizopesoides

XML Treatment for Chorizopesoides guoi

XML Treatment for Chorizopesoides annasestakovae

XML Treatment for Deione

XML Treatment for Deione cheni

XML Treatment for Deione yangi

XML Treatment for Hypsosinga

XML Treatment for Hyposinga pulla

XML Treatment for Mangora

XML Treatment for Mangora baii

XML Treatment for Mangora cephala

XML Treatment for Milonia

XML Treatment for Milonia gemella
